# From structure to clinic: Design of a muscarinic M1 receptor agonist with the potential to treat Alzheimer’s disease

**DOI:** 10.1016/j.cell.2021.11.001

**Published:** 2021-11-24

**Authors:** Alastair J.H. Brown, Sophie J. Bradley, Fiona H. Marshall, Giles A. Brown, Kirstie A. Bennett, Jason Brown, Julie E. Cansfield, David M. Cross, Chris de Graaf, Brian D. Hudson, Louis Dwomoh, João M. Dias, James C. Errey, Edward Hurrell, Jan Liptrot, Giulio Mattedi, Colin Molloy, Pradeep J. Nathan, Krzysztof Okrasa, Greg Osborne, Jayesh C. Patel, Mark Pickworth, Nathan Robertson, Shahram Shahabi, Christoffer Bundgaard, Keith Phillips, Lisa M. Broad, Anushka V. Goonawardena, Stephen R. Morairty, Michael Browning, Francesca Perini, Gerard R. Dawson, John F.W. Deakin, Robert T. Smith, Patrick M. Sexton, Julie Warneck, Mary Vinson, Tim Tasker, Benjamin G. Tehan, Barry Teobald, Arthur Christopoulos, Christopher J. Langmead, Ali Jazayeri, Robert M. Cooke, Prakash Rucktooa, Miles S. Congreve, Malcolm Weir, Andrew B. Tobin

**Affiliations:** 1 Sosei-Heptares, Steinmetz Building, Granta Park, Cambridge, CB21 6DG, UK; 2 The Centre for Translational Pharmacology, Institute of Molecular, Cell and Systems Biology, College of Medical, Veterinary and Life Sciences, University of Glasgow, Glasgow, G12 8QQ, UK; 3 Cross Pharma Consulting Ltd, 20-22 Wenlock Road, London, N17GU, UK; 4 Brain Mapping Unit, University of Cambridge, Department of Psychiatry, Herchel Smith Building, Cambridge, CB20SZ, UK; 5 Eli Lilly & Co, Neuroscience Discovery, Erl Wood Manor, Windlesham, Surrey, GU20 6PH, UK; 6 H. Lundbeck A/S, Neuroscience Research, Ottiliavej 9, 2500 Valby, Copenhagen, Denmark; 7 Center for Neuroscience, Biosciences Division, SRI International, 333 Ravenswood Avenue, Menlo Park, CA, 94025, USA; 8 University Department of Psychiatry, University of Oxford, Warneford Hospital, Oxford, OX12JD, UK; 9 P1vital, Manor house, Howbery Buisness Park, Wallingford, OX108BA, UK; 10 Centre for Cognitive Neuroscience - Duke-NUS Medical School, 8 College Road, 169857, Singapore; 11 Neuroscience and Psychiatry Unit, University of Manchester, Manchester, M139PT UK; 12 Drug Discovery Biology, Monash Institute of Pharmaceutical Sciences and Department of Pharmacology, Monash University, Parkville 3052, Victoria, Australia; 13 ARC Centre for Cryo-electron Microscopy of Membrane Proteins, Monash Institute of Pharmaceutical Sciences, Monash University, Parkville 3052, Victoria, Australia; 14 Protogenia Consulting Ltd, PO-Box 289, Ely, CB79DR, UK

## Abstract

Current therapies for Alzheimer’s disease seek to correct for defective cholinergic transmission by preventing the breakdown of acetylcholine through inhibition of acetylcholinesterase, these however have limited clinical efficacy. An alternative approach is to directly activate cholinergic receptors responsible for learning and memory. The M1-muscarinic acetylcholine (M1) receptor is the target of choice but has been hampered by adverse effects. Here we aimed to design the drug properties needed for a well-tolerated M1-agonist with the potential to alleviate cognitive loss by taking a stepwise translational approach from atomic structure, cell/tissue-based assays, evaluation in preclinical species, clinical safety testing, and finally establishing activity in memory centers in humans. Through this approach, we rationally designed the optimal properties, including selectivity and partial agonism, into HTL9936—a potential candidate for the treatment of memory loss in Alzheimer’s disease. More broadly, this demonstrates a strategy for targeting difficult GPCR targets from structure to clinic.

**Graphical abstract:**

**Figure:**
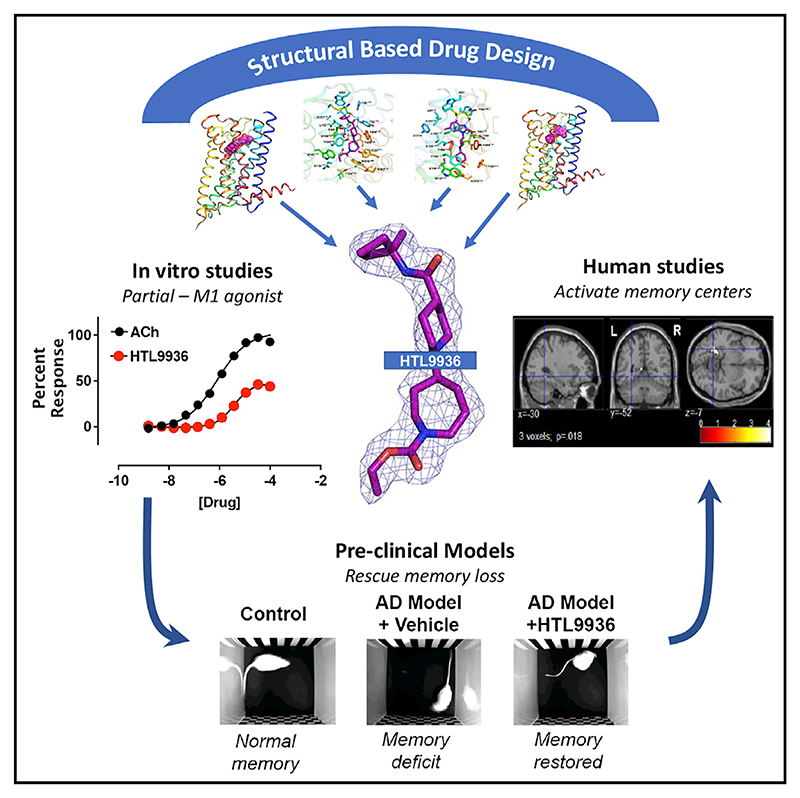

## Introduction

Alzheimer’s disease (AD), the most common cause of age-related dementia, represents one of the most urgent healthcare challenges facing us today with numbers affected projected to increase ~50 million worldwide to 75 million by 2030 in line with an aging global population. The “*cholinergic hypothesis of AD*” postulates that learning and memory deficits result from the loss of cholinergic innervation to the entorhinal cortex and hippocampus from nuclei in the basal forebrain, which may be partially recovered by elevating acetylcholine (ACh) levels, through the inhibition of the ACh catabolising cholinesterases (Bartus et al., 1982; Francis et al., 1999). Restoration of defective cholinergic transmission via inhibition of the cholinesterases is the primary symptomatic treatment for cognitive deficits associated with AD (Douchamps and Mathis, 2017). Despite showing some efficacy, principally in the early stages of disease, cholinesterase therapies show significant dose-related adverse responses, particularly gastrointestinal, that limit clinical use (Courtney et al., 2004; Inglis, 2002; Thompson et al., 2004).

Based on its high expression in areas such as the hippocampus and cortex (Bradley et al., 2017) and the pro-cognitive effects in pre-clinical animal studies (Bradley et al., 2017; Digby et al., 2012; Ghoshal et al., 2016; Moran et al., 2018; Shirey et al., 2009), the M1 muscarinic acetylcholine receptor (M1-receptor), one of five muscarinic receptor subtypes (M1−M5), is widely considered to be a key or mediator of cognitive function and thereby a target for the treatment in AD (Conn et al., 2009a; Conn et al., 2009b; Felder et al., 2018). This has led many pharmaceutical companies to pursue drug discovery programs targeting the M1-receptor (Felder et al., 2018).

A number of orthosteric muscarinic agonists that utilize the binding site of the natural ligand acetylcholine have advanced to the clinic (Felder et al., 2018) and showed tantalising evidence of efficacy. These include the M1/M4 preferring agonist xanome-line that improved both cognitive and behavioral disturbances in AD patients (Bodick et al., 1997a, 1997b) and improved symptoms in schizophrenia patients (Shekhar et al., 2008), and the bitopic M1-ligand GSK1034702 (Bradley et al., 2018; Budzik et al., 2010; Nathan et al., 2013), which improved immediate and delayed recall in a nicotine abstinence study. Both programs were subsequently terminated based on cholinergic-adverse responses, predominantly within gastrointestinal and cardiovascular systems.

The challenge then was to design muscarinic agonists with a pharmacological profile that would activate receptors in the CNS in areas such as the cortex and hippocampus involved in learning and memory while avoiding side effects, particularly gastrointestinal and cardiovascular, that would be unacceptable in a drug for elderly patients. Attempts to map compound pharmacology to physiological activity has proved exceedingly difficult. This is hampered by the lack of selectivity across muscarinic subtypes. An obvious approach therefore would be to design an M1-agonist with high selectivity particularly over M2- and M3-receptors since these are considered to be the primary receptor subtypes mediating peripheral adverse responses. However, in the case of muscarinic receptors, this is very difficult due to the high sequence similarity of the orthosteric binding site across all five receptor subtypes (Vuckovic et al., 2019). Furthermore, it was possible that some of the observed side effects might be mediated through the M1-receptor itself (Alt et al., 2016; Bradley et al., 2020; Davoren et al., 2016). We hypothesized these challenges could potentially be overcome by a partial agonist that might activate highly expressed central receptors while showing reduced activity at peripheral M1-receptors where expression is lower.

Here we describe the strategy taken to use structure-based drug design (SBDD) (Congreve et al., 2017; Jazayeri et al., 2017) to identify a selective M1-receptor orthosteric partial agonist (HTL9936) and to carefully profile the pharmacology of this molecule in a step-wise translational approach *in vitro* and *in vivo* to confirm whether this resulted in the desired profile devoid of the dose-limiting side effects associated with previous M1-receptor agonists. As a final translational step prior to progressing to evaluation in patients with AD, the pharmacology was evaluated in early clinical studies designed to establish the safety profile at doses that produce evidence of functional target engagement in the CNS of healthy volunteers.

## Results

### Screening fragment-like compounds as starting points for drug design

**Figure 1:**
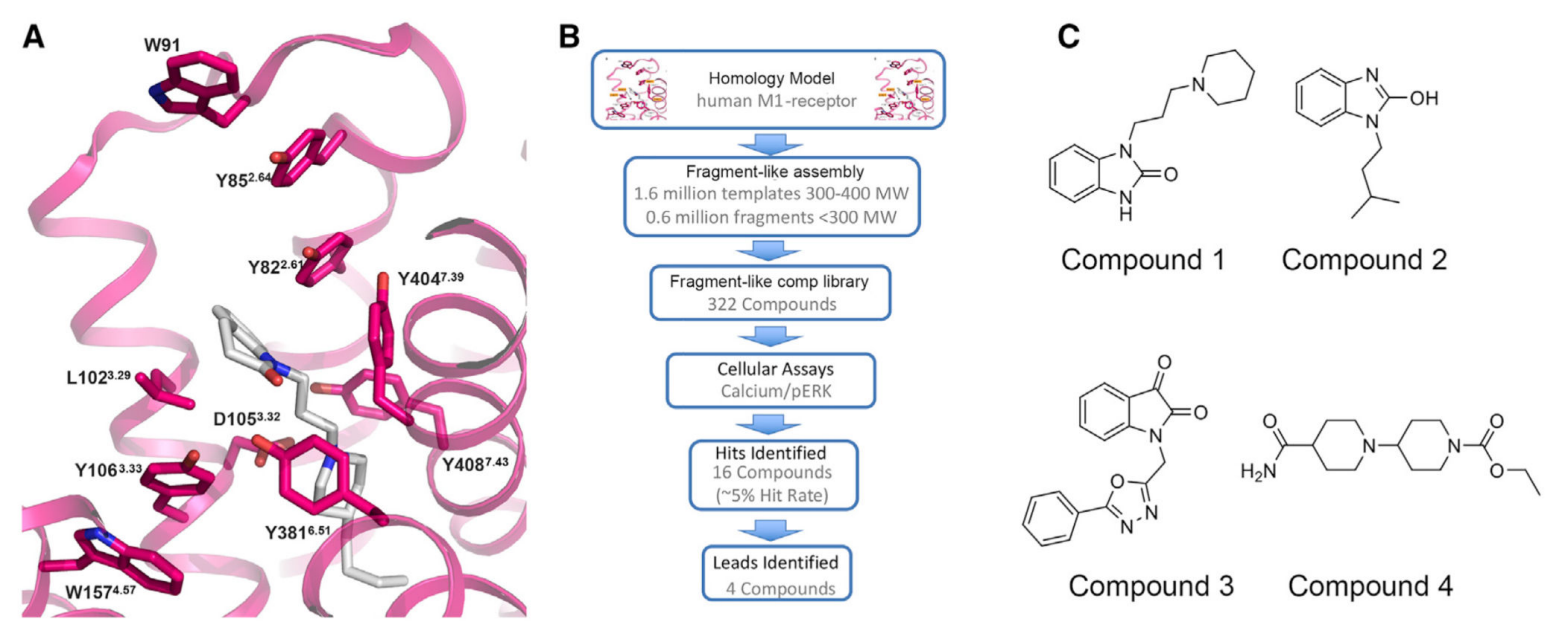
Initial M1-receptor homology model and hit-finding campaign (A) Original homology model of the human M1-receptor with the muscarinic agonist 77-LH-28-1 docked into the orthosteric site used to support identification of a fragment library for hit identification. Ballesteros-Weinstein residue numbering is shown in superscript. (B and C) (B) Schematic of the fragment screening campaign to identify 16 hit compounds possessing M1-receptor activity, from which (C) 4 fragment-like hits (Compounds 1−4) were identified.

To initiate hit finding, homology models of the human M1-receptor bound to the agonist 77-LH-28-1 were derived based on the crystal structure of the avian β_1_-adrenergic receptor:cyanopindolol (PDB: 2VT4) complex (Warne et al., 2008); the best available template at the time. In this model (Figure 1A), residues Y82^2.61^, L102^3.29^, D105^3.32^, and Y408^7.43^ (Ballosteros and Weinstein GPCR residue numbering scheme) lined the orthosteric pocket consistent with mutagenesis data (Lebon et al., 2009). To support hit identification, a careful consideration of predicted binding modes of known agonists were made in the homology model, and a targeted library of fragment-like compounds was subsequently assembled *in silico*. The aim was to enrich for known chemotypes incorporating novel exemplars of privileged ring systems and functional groups that were observed across different classes of muscarinic agonist ligands (Avlani et al., 2010; Budzik et al., 2010; Goodwin et al., 2007; Lebon et al., 2009). A set of 322 commercially available fragment-like molecules were ultimately purchased following prioritisation by docking molecule selections into the M1 homology model and then tested in an *in vitro* functional extracellular regulated protein kinase-1/2 phosphorylation (pERK1/2) and calcium mobilisation assays (Figure 1B). Sixteen compounds exhibited activity, the top four of which were prioritised for further pharmacological characterization and medicinal chemistry optimization (Figure 1C; Table S1). Of these four hits the bis-piperidine primary amide, Compound 4, demonstrated an EC_50_ of 3.5 μM at the M1-receptor and was considered an attractive starting point for further optimization.

### High-resolution structures of the agonist-bound M1-receptor

To accelerate SBDD we sought to generate a high-resolution crystal structure of the M1-receptor in an agonist-bound conformation. To facilitate structure determination, a thermostabilized receptor construct (termed M1-StaR) (Doré et al., 2011; Robertson et al., 2011) was generated in the presence of the agonist 77-LH-28-1 (Langmead et al., 2008). The M1-StaR contained twelve amino acid substitutions (Figures S1A and S1B) including a tryptophan to alanine substitution at position 101 (W101A^3.28^) that was predicted to enable direct access of small molecule agonists, including 77-LH-28-1, to the orthosteric binding site of the M1-receptor (Lebon et al., 2009). With the exception of W101A^3.28^, none of the other eleven amino acid substitutions were predicted to directly influence the acetylcholine binding site (Figures S1A and S1B). To aid expression, the first 87 amino acids of the M1-receptor were replaced by residues 1−95 of the human M4-receptor. To promote crystalisation, the last 22 residues of C-termini were removed and residues R220−F355 in the third intracellular loop were replaced with T4-lysozyme (T4L) (Figures S1A and S1B). Consistent with an agonist-like conformation, the M1-StaR-T4L had high agonist (77-LH-28-1) binding affinity while showing a significant reduction in antagonist (NMS) affinity compared to wild-type receptor (Table S2).

**Figure 2:**
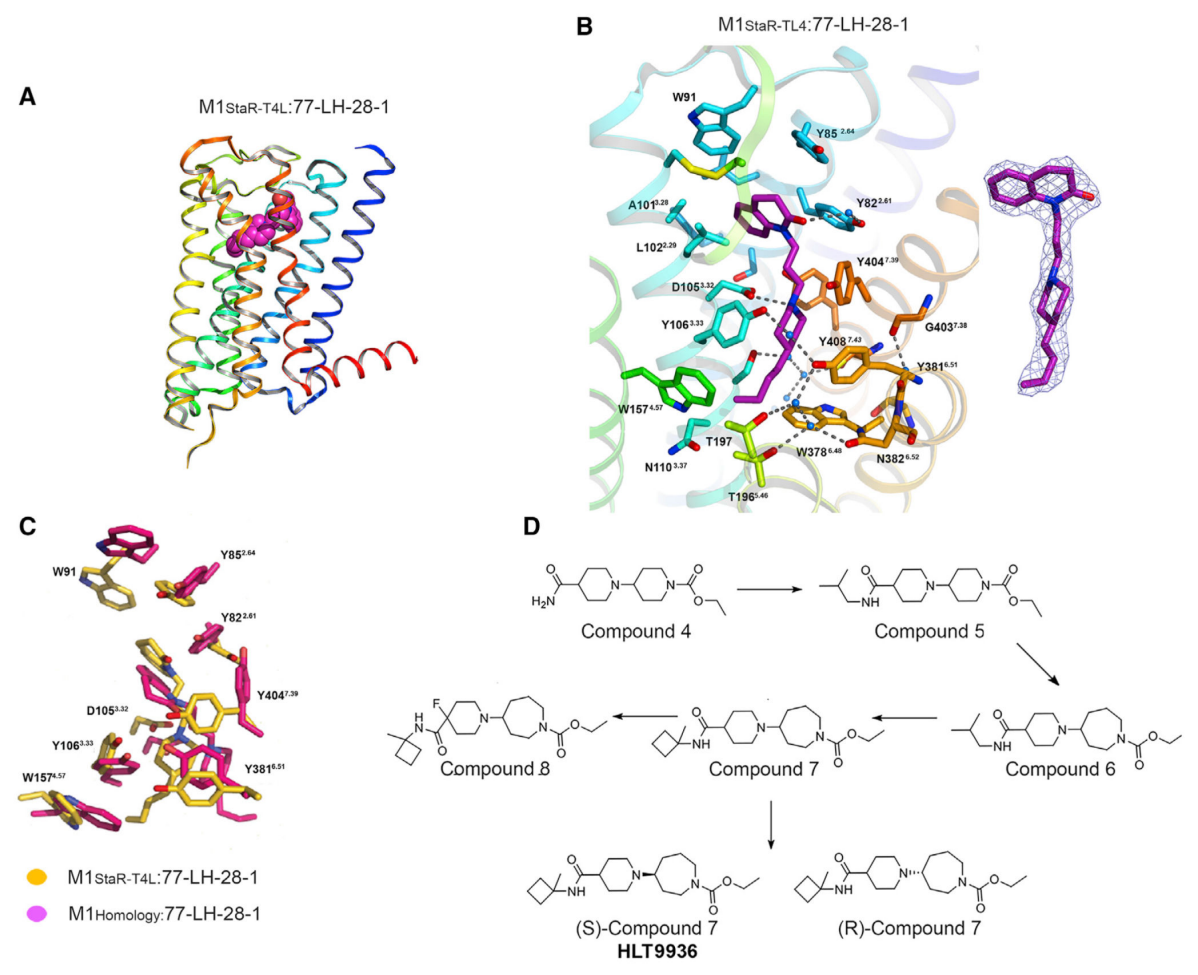
Structure of 77-LH-28-1 bound to the M1-receptor and design of HTL9936 (A) Crystal structure of M1-StaR-T4L shown as ribbons colored blue (N terminus) to red (C terminus), with 77-LH-28 bound in the orthosteric site represented as a space-filled model. (B) Zoomed-in view of the M1-StaR-T4L orthosteric site with 77-LH-28-1 as well as side chains of binding site residues within 5 Å of the ligand, shown as sticks. Water molecules are represented as blue spheres, and hydrogen bonding networks are shown as dashed lines. The 1s contoured 2mFo-dFc electron density map corresponding to 77-LH-28-1 (represented as sticks) is shown as a blue mesh in the right-hand side inset. (C) Superposition of binding site residues of the M1-receptor homology model (pink) onto the crystal structure of the M1-StaR-T4L bound to 77-LH-28-1 (yellow). Ligands for each of these structures are represented as sticks in pink and yellow respectively for the homology model and for the crystal structure and provide a visual summary of the accuracy of the initial homology model. (D) Medicinal chemistry iterations leading to the design of (S)-Compound 7 (HTL9936) from the original hit molecule Compound 4. See also Figures S1 and S2.

The M1-StaR-T4L was subsequently crystallized bound to 77-LH-28-1 in lipidic cubic phase (LCP) and the structure determined to 2.17 Å resolution (Figure 2A; Table S3). In this structure, 77-LH-28-1 is very well defined in the electron density map and is primarily held in place via the charge-charge interaction from the protonated nitrogen within the piperidine fragment at the center of the ligand to the negatively charged aspartate (D105^3.32^) side chain (Figures 2B and 2C; Figures S2A and S2B). This piperidine ring system breaks apart the “tyrosine cage” seen in subsequently published muscarinic antagonist structures (Thal et al., 2016). Toward the apical side of the receptor, the tetrahydroquinoline-2-one moiety of the ligand makes hydrophobic contacts with W91^23.50^, L102^3.29^, Y85^2.64^, and Y82^2.61^ as well as with the disulphide bridge between cysteine residues C98^3.25^ - C178^45.50^, thereby linking transmembrane domain 3 (TM3) extracellular loop 2 (ECL2). The tetrahydroquinoline-2-one oxygen further makes a water-mediated hydrogen bond with the hydroxyl group of Y82^2.61^. The lower part of the 77-LH-28-1, encompassing the piperidine ring and the aliphatic tail, is sheathed within an elongated cavity, delineated by water molecules which are stabilized by an intricate hydrogen-bonding network, and by Y106^3.33^, Y408^7.43^, W157^4.57^, and Y404^7.39^, all of which make van der Waals contacts with the ligand. This structure thus provided important initial co-ordinates to support M1-receptor SBDD.

### Structure-based drug design of a lead M1-receptor agonist—HTL9936

The key challenge we initially sought to address within the hit series was early evidence for M1-receptor selectivity against the highly homologous M2- and M3-receptors. To address this challenge, a series of molecules were synthesized to investigate the structural activity relationship (SAR) among which were a series of small amides including the iso-butyl amide Compound 5 (Figure 2D), which showed improved M1-receptor agonist activity (EC_50_ 316 nM), but still contained weak M2- and M4-receptor activity (Table S4). Analysis of the docked structure of Compound 5 predicted that the shape of the ring system attached to the ethyl carbamate and subsequent structural re-arrangements of the “tyrosine cage” would confer improved selectivity over M2- and M3-receptor subtypes. This was confirmed by the introduction of an azepine ring that filled a sub-pocket of the orthosteric site defined between Tyr106^3.33^, Trp378^6.48^, Tyr381^6.51^, and Cys407^7.42^ in a more efficient manner to give racemic Compound 6 (Figure 2D), which showed sub-micromolar potency at the M1-receptor in calcium assays (EC_50_ 79 nM) with no detectable agonist activity at M2- and M3-receptors and a ten-fold lower potency at M4-receptors (EC_50_ 794 nM) (Table S4). This formed a critical breakthrough in the development of selective M1-receptor agonists.

Further investigation of the structural activity relationships of the amide group determined that lipophilic secondary amides were preferred (e.g., Compounds 14−18; Table S4) while alkyl tertiary amides (e.g., Compounds 19 and 20; Table S4) and alkyl amides with polar substituents (e.g., Compounds 21, 22, and 26; Table S4) were not. An example of a preferred secondary amide is racemic Compound 7 (Figure 2D), which contains a cyclobutylmethyl secondary amide. Separation of racemic Compound 7 gave the enantiomers (S)-Compound 7 (HTL9936) [M1 EC_50_ 32 nM, M2 > 20 μM, M3 > 20 μM, M4 398 nM] and (R)-Compound 7 [M1 EC_50_ 398 nM, M2 > 20 μM, M3 > 20 uM, M4 2.5 μM] (Table S4). The (S)-enantiomer is the preferred chiral center on azepine ring, and (S)-Compound 7 (HTL9936) was progressed to further structural studies and preclinical assessment (Patent:WO2013/072705).

### Structure of the M1-receptor:HTL9936 complex in comparison to other agonists

**Figure 3:**
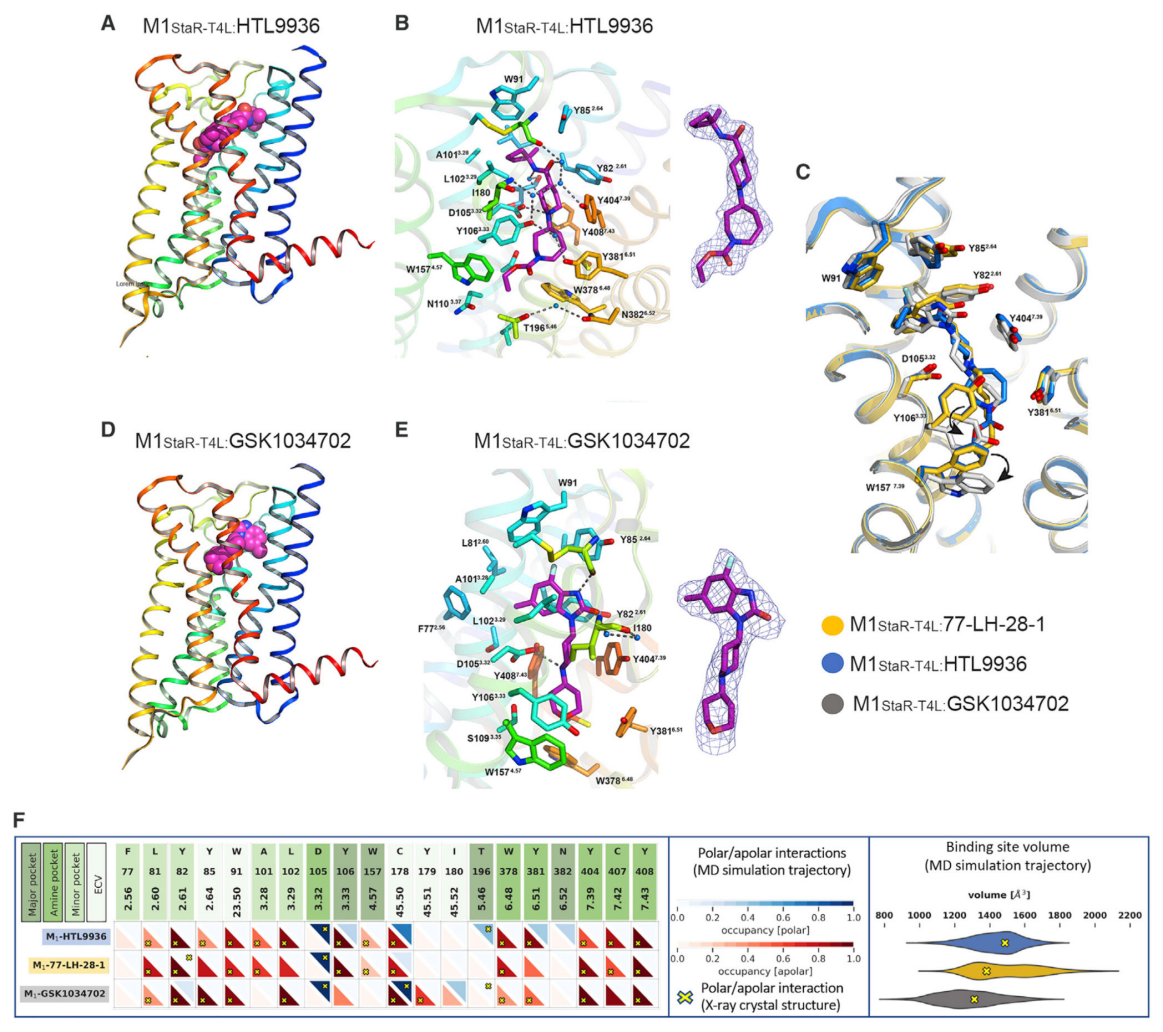
Structural comparison of the active state of the agonist-bound human M1-receptor (A) Crystal structure of the M1-StaR-T4L bound to HTL9936. (B) Ligand binding site of M1-StaR-T4L bound to HTL9936. (C) Superposition of ligand binding site of M1-StaR-T4L bound to HTL9936, GSK1034702 and 77-LH-28-1. In the M1-StaR-T4L-GSK1034702 complex, Y106^3.33^ and W157^4.57^ adopt clearly distinctly rotameric states from similar residues in the other two structures, as indicated by the curved arrows. (D) Crystal structure of the M1-StaR-T4L bound to GSK1034702. (E) Ligand binding site of M1-StaR-T4L bound to GSK1034702. (F) Comparative analysis of structural protein-ligand interactions and ligand binding site volumes of crystal structures and Molecular Dynamics (MD) simulations of M1-StaR-T4L bound to HTL9936, 77-LH-28-1 and GSK1034702. Apolar and polar protein-ligand interactions in crystal structures (yellow crosses) and MD simulation ensembles (red and blue triangles) are defined per amino acid residue as described in the STAR Methods section, including the consideration of water-mediated polar interactions. Major pocket, amine pocket, minor pocket, and extracellular vestibule (ECV) residues are color coded as defined for aminergic GPCR ligand binding site regions (Vass et al., 2019). Binding site volumes of crystal structures (yellow arrows) and MD simulation trajectories (violin plots), for HTL9936 (blue), 77-LH-28-1 (orange) and GSK1034702 (gray) are shown on the right. See also Figures S2 and S3.

To refine our understanding of ligand binding modes driving selectivity at the M1-receptor, we obtained structures of the M1-StaR-T4L with HTL9936 and the M1-agonist GSK1034702 (Budzik et al., 2010); the latter reached early clinical development but was subsequently withdrawn due to dose-limiting cardiovascular and gastrointestinal side effects (Felder et al., 2018). When it was tested alongside other compounds, we observed significant M2-receptor agonist activity for GSK1034702 (Table S4) that may underlie some of the dose-limiting clinical effects and provides a rationale for a direct structural comparison. In our comparative analysis of different M1-receptor-ligand binding modes, we used previously defined aminergic GPCR ligand binding site regions (Vass et al., 2019) including the centrally located amine pocket (D103^3.32^, S109^3.36^, W378^6.48^, Y381^6.51^, Y404^7.39^, C407^7.42^, Y408^7.43^), connecting the major pocket (Y106^3.33^, N110^3.37^, W157^4.56^, A^5.43^, T196^5.46^, N382^6.52^) and minor pocket (F77^2.56^, L81^2.60^, Y282^2.61^, A/W101^3.28^, L102^3.29^), extending toward the extracellular vestibule (Y85^2.64^, W91^23.50^, C178^45.50^, I180^45.52^). We found that like 77-LH-28-1, HTL9936 was primarily held in place via the charge-charge interaction from the protonated nitrogen to the negatively charged aspartate, D105^3.32^ in the amine pocket (Figures 3A−3C; Figure S2C and S2D). HTL9936 demonstrates a similar re-arrangement of the tyrosine cage to 77-LH-28-1 with nitrogen of the piperidine ring making contact with Y404^7.39^ causing a rotation that confers a unique conformation of another tyrosine within the cage, Y381^6.51^ (Figures 3A−3C). The base of the amine pocket is formed by C407^7.42^, making hydrophobic contacts to the homopiperidine ring, and W378^6.48^, which is forming an edge-to-face π-stacking arrangement with the delocalized π electrons of the carbamate system.

GSK1034702 is primarily held in place via a similar interaction with the negatively charged aspartate D105^3.32^ (Figures 3C−3E; Figures S2E and S2F). However, a number of notable differences were observed in the binding mode of GSK1034702 compared to 77-LH-28-1 and HTL9936, which despite the identical amino acid sequences between the muscarinic subtypes in the orthosteric site can rationalise the reduced selectivity versus M2-receptor agonism. Most notably, the reduced size of the tetrahydropyran fragment at the base of the ligand enables direct contact with Y106^3.33^ that in turn is associated with displacement of W157^4.57^, W378^6.48^, and Y404^7.39^, thereby forcing a distinct orientation in this region of the binding pocket from that seen in the structures with 77-LH-28-1 and HTL9936. In addition, at the top of the minor binding pocket the larger 7-fluoro-5-methyl-benzimidazol-2-one portion of GSK1034702 occupies the whole of the region vacated by the W101^3.28^A (W101A) mutation in the M1-StaR.

Comparative MD simulations were performed in the M1-StaR background to provide complementary insights into the flexibility of M1-ligand binding mode conformations and the stability of water-mediated polar interaction networks identified from the structural analysis (Figure 3F). HTL9936 forms stable water-mediated polar interaction networks in the major pocket with Y106^3.33^, T196^5.46^, Y381^6.51^, and N382^6.52^ via its carbamate moiety and in the minor pocket and extracellular vestibule with C178^45.50^ and Y404^7.39^ via its amide group. Similarly, GSK1034702 forms stable water-mediated polar interaction networks with its tetrahydro-*2H*-puran moiety and Y106^3.33^ and T196^5.46^ and between its benzimidazol-2-one group and Y82^2.61^, I180^45.52^ and Y381^6.51^. In contrast, the water-mediated polar interaction between 77-LH-28-1 and Y82^2.61^ observed in the crystal structure is not stable in MD simulations. The pentyl moiety of 77-LH-28-1 is flexible, adopting alternative binding modes between Y106^3.33^/W157^4.57^ or between A193^5..43^/Y381^6.51^ in the major pocket. The broad range of binding site volumes sampled in MD simulations (Figure 3F) is consistent with the relative flexibility of the M1-receptor:77-LH-28-1 complex compared to GSK1034702 and HTL9936. Analysis of the binding site volumes for the partial agonist HTL9936 and agonists 77-LH-28-1 and GSK1034702 highlight a closer alignment to the binding site volumes of the antagonist tiotropium than the larger binding site volumes sampled for the small agonist iperoxo (Figure S3). Altogether, the comparative analysis of M1-receptor ligand interaction fingerprints (Kooistra et al., 2015; Kooistra et al., 2016) and structurally resolved ligand binding sites in the M1-StaR indicate that the partial agonist HTL9936 has several unique M1-receptor binding mode features compared to agonists 77-LH-28-1 and GSK1034702 (Figure 3). HTL9936 has a more extended, stable binding mode targeting a larger M1-receptor binding site connecting major and minor pockets, stabilized by the novel, optimized piperidine-azepine ring system of HTL9936. In addition, the carbamate moiety of HTL9936 stabilizes a unique, extended water mediated polar receptor-ligand interaction network in the major pocket between TM3, TM5, and TM6. While keeping in mind that the MD simulations were performed in the M1-StaR background and not the WT receptor, the combined structural features of HTL9936 described here potentially provide a basis for the partial agonism of this novel M1-receptor modulator.

### *In vitro* pharmacological and signaling characterization of HTL9936

**Figure 4:**
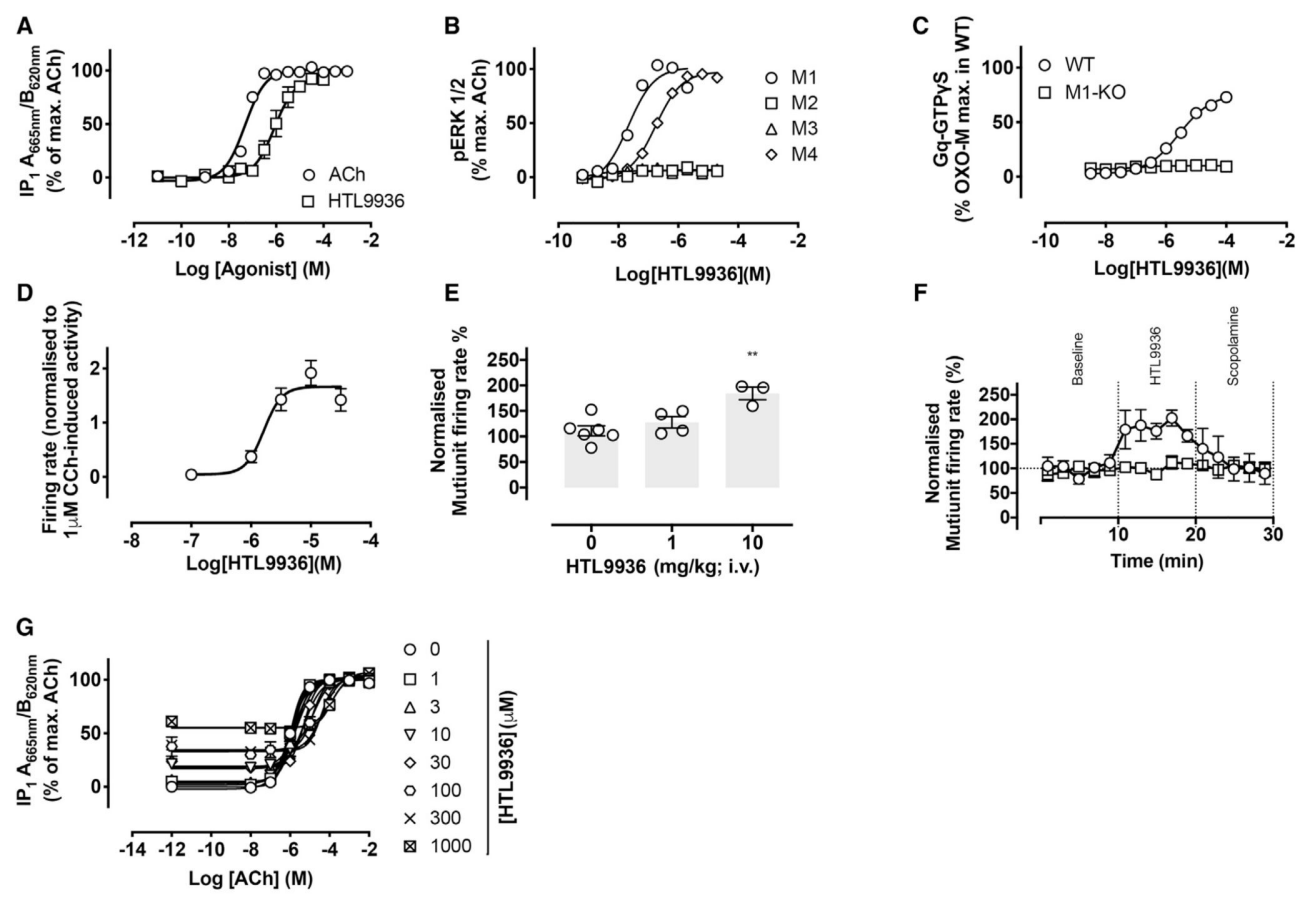
*In vitro* pharmacological characterization of HTL9936 (A) Inositol phosphate accumulation elicited by ACh or HTL9936 via the human M1-receptor expressed in CHO Flp-In cells. Data are expressed as means ± SEM of 3−4 independent experiments performed in duplicate. (B) ERK1/2 phosphorylation elicited by HTL9936 at the M1-, M2-, M3-, or M4-receptors expressed in CHO cells. Data are expressed as a percentage of the maximum response stimulated by ACh and are means ± SEM of 2-13 experiments performed in duplicate. (C) Stimulation of [^35^S]-GTPyS binding to cortical membranes prepared from wild-type (WT) or M1-knockout mice (M1-KO). Data shown are means ± SEM of 3 experiments (pEC_50_ = 5.6 ± 0.1 at the WT). (D) HTL9936 dose-dependently increases CA1 neurons spontaneous firing. Data are expressed as mean firing rates (normalized to carbachol effect) over the 10 last min of each compound exposure period ± SEM. (E) Summary statistics of firing rate of CA1 neurons recorded *in vivo* in isoflurane anaesthetised rats compared to vehicle-treated animals calculated as the 5 min average after vehicle, 1 mg/kg or 10 mg/kg HTL9936 treatment. (F) 10 mg/kg i.v. administered HTL9936 (open circles) produced a significant increase in the *in vivo* firing rate of CA1 neurons compared to vehicle treated animals (squares). The muscarinic antagonist scopolamine (dose 1 mg/kg) administered i.v. after 10 min reversed the increase in firing back to baseline activity but had no effect on vehicle-treated animals. Data shown are mean of 3 rats expressed as percent of pre-drug baseline. (G) HTL9936 antagonism of ACh-stimulated inositol phosphate accumulation in CHO Flp-In cells expressing the human M1-receptor. Cells were incubated with 3 μM phenoxybenzamine prior to addition of HTL9936 at escalating concentrations. Data are means ± SEM of 3−4 independent experiments performed in duplicate. See also Figures S4 and S5.

*In vitro* profiling in CHO cells overexpressing human M1-receptor confirmed the agonist activity of HTL9936 in pERK1/2 (EC_50_ 32 nM), label-free dynamic mass redistribution (EC_50_ 79 nM) and inositol phosphate (IP1) assay formats (EC_50_ 631 nM; Figures 4A and 4B; Table S5). Importantly, HTL9936 demonstrated no detectable agonism at human M2-, M3-, or M5-receptors, although partial agonist activity was observed at the human M4-receptor (Table S5). HTL9936 was further confirmed to demonstrate similar pharmacological activity across human, dog, rat, and monkey M1-receptors (Table S5). Equilibrium radioligand binding studies using the antagonist [^3^H]-NMS demonstrated that like acetylcholine, HTL9936 had weak affinity for the human M1 (pKi = 4.7 ± 0.03, n = 4), M2 (pKi = 5.5 ± 0.3, n = 6), and M4 (pKi = 5.4, n = 1) receptors while there was no detectable binding to the M3-receptor.

The recombinant overexpressed cells provide a sensitive assay for muscarinic agonism—however, demonstrating agonist activity in native systems increases confidence in achieving the desired pharmacological profile. This is particularly important in the case of partial agonists. In membranes prepared from the cortex of wild-type mice, HTL9936 stimulated a robust increase in G_q/11_ protein-coupling (EC_50_ = 2.5μM, E_max_ 76% of the oxotremorine-M response [n = 3]), which was absent in membranes prepared from M1-receptor KO animals (Figure 4C). Electrophysiological recordings in rat hippocampal slices established that HTL9936 increased the intrinsic excitability and spontaneous firing rates of CA1 pyramidal cells with a EC_50_ = 1.6μM (Figure 4D). These findings were corroborated using *in vivo* electrophysiological recordings in adult rats where HTL9936 caused a concentration-dependent increase in neuronal firing rate (Figure 4E) that was reversed with the muscarinic antagonist scopolamine (i.v.) (Figure 4F).

While HTL9936 was observed to behave as a full agonist at the human M1-receptor in both pERK1/2 and DMR recombinant assay formats (Table S5), it appeared as a partial agonist at the rat receptor (Table S5) and in native mouse cortical membranes in GTPγS assays (Figure 4C). To resolve this apparent contradiction, we determined the degree of intrinsic efficacy of HTL9936 relative to acetylcholine by using the receptor-alkylating agent phenoxybenzamine (PBZ) to irreversibly reduce M1-receptor levels in the cell line recombinantly overexpressing the human M1-receptor. Inactivation of M1-receptors with PBZ resulted in a reduction of both potency and maximal responses with HTL9936 more sensitive than acetylcholine to these effects (Figure S4). Moreover, under equivalent conditions of receptor depletion, HTL9936 behaved as a partial agonist in interaction assays with acetylcholine, eliciting a rightward shift in the IP_1_ acetylcholine concentration-response curve with increasing concentrations of HTL9936 (Figure 4G). Analysis of the data yielded Schild slopes approximating to unity (0.95 ± 0.08) and a pA_2_ value of 5.2 ± 0.11; that correlated with the pKi for HTL9936 described in radio-ligand binding studies above.

We next assessed the potential of HTL9936 to act as a biased ligand directing M1-receptor signaling toward one signaling pathway in preference to another (Smith et al., 2018). We determined the transduction co-efficient (t) for acetylcholine (the reference ligand) and HTL9936 coupling the M1-receptor to G_q_, G_11_, G_15_, the inositol phosphate (IP) pathway, pERK, and arrestin recruitment to the plasma membrane and receptor-internalisation (Figures S5A−S5H). By determining the ratios of the transduction coefficients (ΔΔlog(τ/K_A_)) (Kenakin and Christopoulos, 2013), we observe very little if any bias activity of HTL9936 (all ΔΔlog(τ/K_A_) values are between 1 and −1, indicating that HTL936 performed as a relatively unbiased agonist of the M1-receptor (Figure S5H)). These experiments also showed the partial agonism of HTL9936 (Figures S5A−S5G).

HTL9936 was also found to be highly selective against a broad panel of 62 GPCRs (DiscoverX neurological and psychiatric panel) where at 10 μM, HTL9936 exhibited <25% agonism at other receptors.

### Evaluation of HTL9936 in pre-clinical efficacy models

The *in vivo* pharmacology of HTL9936 was examined across a range of efficacy models in mice, rats, dogs, and non-human primates as described in the following sections.

Prior to testing in behavioral models, HTL9936 brain:plasma and brain:CSF ratios were measured in rats after intravenous (1 mg/kg) and oral (10 mg/kg) dosing and revealed significant distribution into the CNS (measured by total brain concentration and calculated unbound drug; Table S6). The CNS distribution profile was confirmed in dogs where assessment of the distribution into the CSF after subcutaneous dosing demonstrated CSF:plasma ratios of 0.66, 0.6, and 0.65 at the 0.3, 1, and 2 mg/kg doses (Table S6). A Kp,uu of ~0.2 was calculated in mice. The calculated, or measured, plasma and brain exposures achieved in these studies and the approximate unbound drug levels as a ratio of the *in vitro* potency values determined for HTL9936 are provided in Table S6.

**Figure 5:**
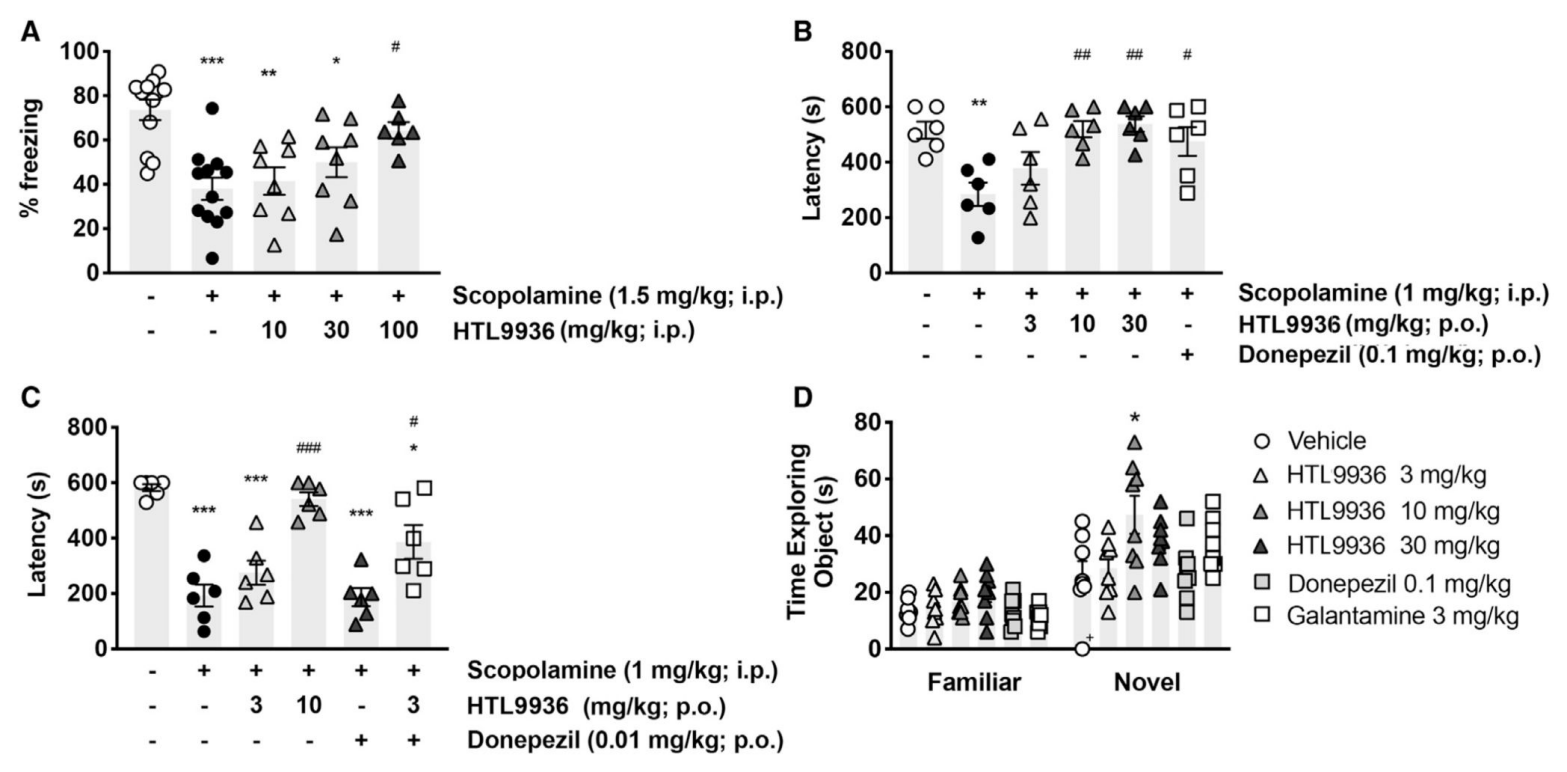
*In vivo* pharmacological characterization of HTL9936 in rodents (A) Effects of HTL9936 (10, 30, or 100 mg/kg; i.p.) on scopolamine (1.5 mg/kg; i.p.)-induced impairments in contextual fear conditioning in male C57BL/6J mice. Data are expressed as means ± SEM of 6−12 mice. Data were analyzed using one-way ANOVA with Bonferroni’s multiple comparison test, where *p < 0.05, **p < 0.01, ***p < 0.001 versus vehicle alone and ^#^p < 0.05 versus 1.5 mg/kg scopolamine-treated. (B and C) Effects of HTL9936 (3, 10, or 30 mg/kg; p.o.) alone (B) or in combination with donepezil (C) on scopolamine (1 mg/kg; i.p.)-induced amnesia in a passive avoidance paradigm in adult Wistar rats. HTL9936 or donepezil (0.1 or 0.01 mg/kg) were administered 90 min prior to the training period. Data shown are means ± SEM of 6 rats. Data were analyzed using a one-way ANOVA where *p < 0.05, **p < 0.01, ***p < 0.001 versus vehicle alone and ^#^p < 0.05, ^##^p < 0.01, ^###^p < 0.001 versus 1 mg/kg scopolamine-treated. (D) Effects of acute HTL9936 (p.o.) administration on improvement of memory performance in a rodent novel object recognition paradigm. Adult male Wistar rats were treated with vehicle (saline) or HTL9936 (3, 10, or 30 mg/kg) 90 min prior to training. Galanthamine (3 mg/kg) or donepezil (0.1 mg/kg) administered 60 min prior to training were used as positive controls. Time (s) spent exploring the novel object during the testing phase is shown. Data shown are means ± SEM of 8 rats (one animal highlighted with an (+) did not respond in the vehicle group and was removed from the analysis). Data were analyzed using one-way ANOVA with Dunnett’s multiple comparison test, where *p < 0.01 comparing treatment versus vehicle. See also Figure S6 and Tables S5 and S6.

Initial assessments of HTL9936 in cognitive tasks focused on reversal of learning and memory deficits induced by the non-specific muscarinic antagonist scopolamine. In a mouse model of fear conditioning involving associative learning and activation of hippocampal CA1, medial PFC, and basolateral amygdala (Maksymetz et al., 2019), administration of scopolamine (i.p, 1.5 mg/kg) 30 min prior to fear conditioning training resulted in a significant reduction in contextual-dependent fear learning and memory (Figure 5A). Co-administration of HTL9936 with scopolamine resulted in a dose-dependent restoration of learning and memory responses (Figure 5A). Next, the effects of HTL9936 were examined in a rat passive avoidance test, a spatial memory consolidation task dependent on intact entorhinal-hippocampal interactions (Myhrer, 2003) where animals learn to avoid making a choice that results in receiving a mild aversive stimulus. Treatment with scopolamine (1 mg/kg i.p. 6 h post-training) induced a statistically significant amnesic effect that was reversed in a dose-dependent manner by HTL9936 (Figure 5B; estimated concentration of unbound drug in the brain [Cu,br] = 262 and 786 nM at 10 and 30 mg/kg doses p.o, respectively). The maximal response observed with HTL9936 was equivalent to that obtained using the clinically approved acetylcholinesterase inhibitor donepezil (0.1 mg/kg; p.o) (Figure 5B). In control experiments, HTL9936 demonstrated no difference in effect in open-field exploratory behavior consistent with unaltered locomotor, anxiolytic, and general behavior of the animals (Figure S6).

Learning and memory deficits in AD have been attributed to a loss of cholinergic transmission that are treated clinically with acetylcholinesterase inhibitors (e.g., donepezil) (Bartus et al., 1982; Francis et al., 1999). Therefore, it was important to assess the potential effects of combining HTL9936 with the current clinical standard of care, donepezil. For these studies, we employed a dose of donepezil (0.01 mg/kg) that was inactive in the rat passive avoidance model combined with a dose of HTL9936 of 3mg/kg (estimated Cu,br = 79 nM) that similarly gave no response alone (Figure 5C). When combined, HTL9936/donepezil resulted in a significant partial reversal of scopolamine-induced deficits consistent with an additive-like effect between these compounds (Figure 5C).

In experiments not involving scopolamine, treatment with HTL9936 improved working memory in the rat novel object recognition paradigm that reflects improved functioning of fronto-hippocampal and entorhinal circuits (Cohen and Stackman, 2015). The effective dose of HTL9936 was 10 mg/kg (estimated Cu,br = 262 nM) (Figure 5D), which showed an effect that was significantly better than the positive controls donepezil and another acetylcholinesterase inhibitor, galantamine (Figure 5D).

Finally, compounds which stimulate CNS activity in particular dopaminergic function are associated with increases in locomotor activity. Such activity can compromise interpretation of cognitive measure. HTL9936 at doses up to 100 mg/kg showed no adverse responses nor had any effect on locomotor activity when administered alone or in combination with amphetamine. The amphetamine-induced hyper-location model is also known to be sensitive to the effects of M4-receptor agonists and therefore, despite the evidence of M4 activity in the recombinant assays HTL9936, at least in this model, did not appear to demonstrate evidence of M4-receptor agonist-like activity.

### HTL9936 reversed cognitive deficits in a mouse model of neurodegeneration

**Figure 6:**
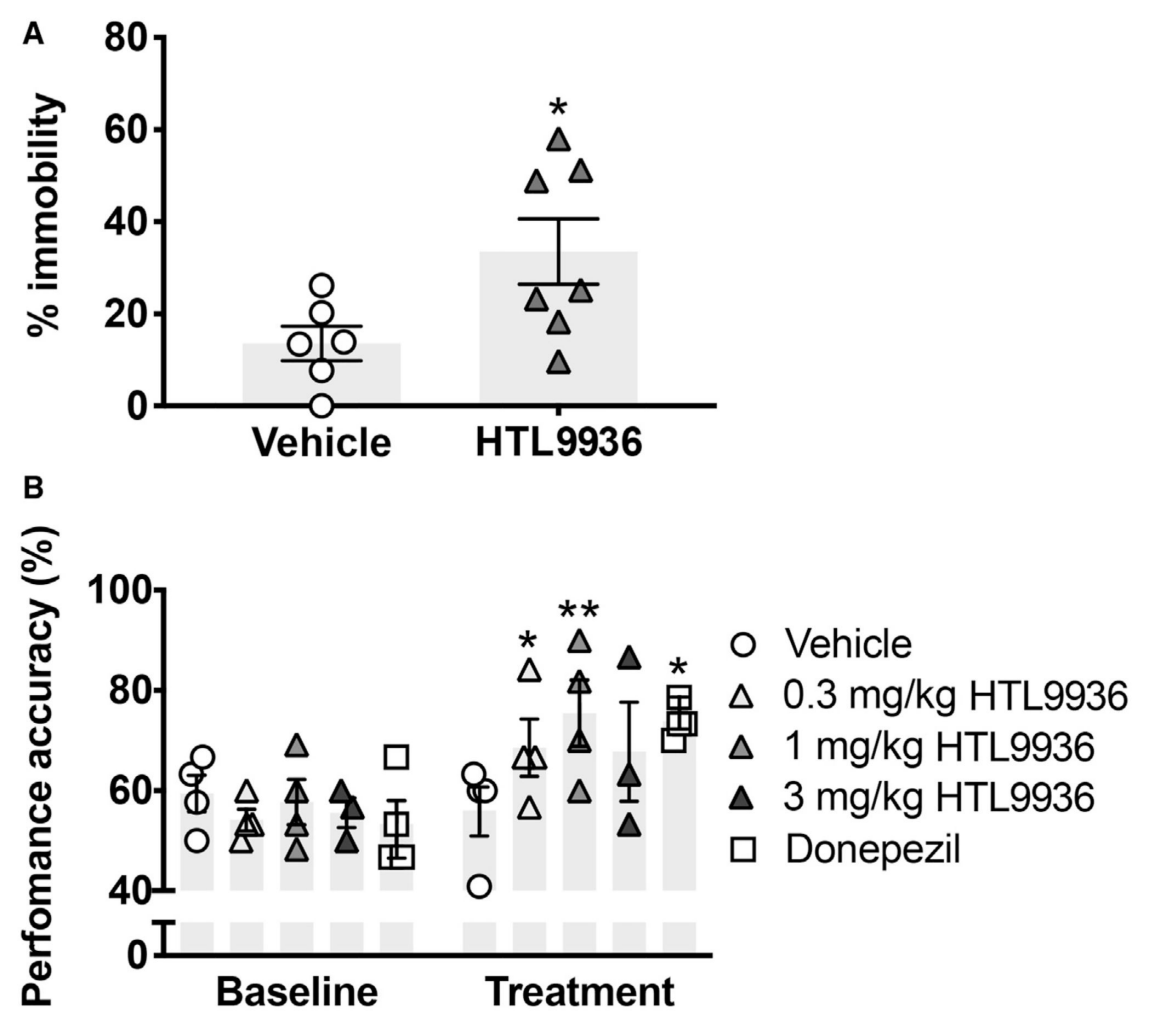
*In vivo* effects of HTL9936 in neuro-degenerative backgrounds (A) Effects of HTL9936 on improvement of fear conditioning learning and memory deficits in prion-diseased mice. Data shown represent immobility levels during the context retrieval phase in prion-infected mice treated with vehicle (5% glucose) or HTL9936 (30 mg/kg; i.p.) 30 min prior to fear conditioning training. Data represent means ± SEM of 6−7 mice and were analyzed using a Student’s t test where *p < 0.05. (B) Effects of HTL9936 administration on cognitive function of aged beagle dogs in the DNMP test. Data shown are DNMP performances at the 55 s delay in the lowest performing subjects at baseline and following 10−11 days treatment with vehicle (0.9% saline), HTL9936 (0.3, 1 and 3 mg/kg; s.c.), or 1.5 mg/kg donepezil (p.o.). Mean DNMP performance was calculated for the 5 baseline DNMP sessions and the last 5 treatment DNMP session. Data shown are means ± SEM and data were analyzed using a two-way ANOVA with Dunnett’s multiple comparison test where *p < 0.05 versus vehicle. See also Tables S5 and S6.

We next wanted to test the effects of HTL9936 in the context of neurodegenerative disease. Our previous studies have determined that murine prion disease is associated with disrupted hippocampal cholinergic innervation that results in learning and memory deficits that could be restored by donepezil and muscarinic ligands (Bradley et al., 2017). Consistent with the notion that HTL9936 can restore cholinergic tone in a manner that might be relevant in the treatment of cognitive dysfunction in AD, we found that HTL9936 significantly improved fear conditioning learning and memory in murine prion disease (Figure 6A). In these studies, Tg37 hemizygous mice at 9 weeks post inoculation (w.p.i.) with Rocky Mountain Laboratory (RML) prions were treated with HTL9936 (30 mg/kg; i.p.) 30 min prior to the training phase. On re-exposure to the training environment 24 h later, mice treated with HTL9936 showed significantly higher immobility levels (~34%) than mice receiving vehicle (~13%), indicating that hippocampal-dependent contextual learning and memory processes in neurodegenerative mice were improved by acute administration of HTL9936 in this disease model (Figure 6A).

### HTL9936 demonstrates cognitive benefits in aged beagles

Similar to clinical AD pathology, beagle dogs exhibit age-dependent cognitive decline, Aβ pathology, and evidence of cholinergic hypofunction, suggesting that this may represent a valuable pre-clinical species with translational relevance for muscarinic agonists targeting cognitive decline (Araujo et al., 2011). A delayed non-matching to position (DNMP) cognitive model was used to assess the effects of HTL9936 on a visual-spatial working memory task in aged beagle dogs. HTL9936 was administered subcutaneously at 0.3, 1, and 2 mg/kg. Bioanalysis of plasma and CSF at 4 h post-dose confirmed plasma:CSF ratios were consistent with pre-clinical rodent assessment (measured C_csf_ concentrations = 13, 48, and 128 nM, respectively; see Table S6). Baseline DNMP performance was assessed daily for 5 days followed by daily dosing of HTL9936 or vehicle for 10 days with DNMP assessment on 5 consecutive occasions. Significant improvements in visual-spatial memory were seen after treatment with HTL9936 at 0.3 mg/kg and 1 mg/kg. The effects at the 1 mg/kg dose of HTL9936 were equivalent to that of donepezil (Figure 6B).

### Translation of muscarinic receptor agonism to non-human primates

**Figure 7:**
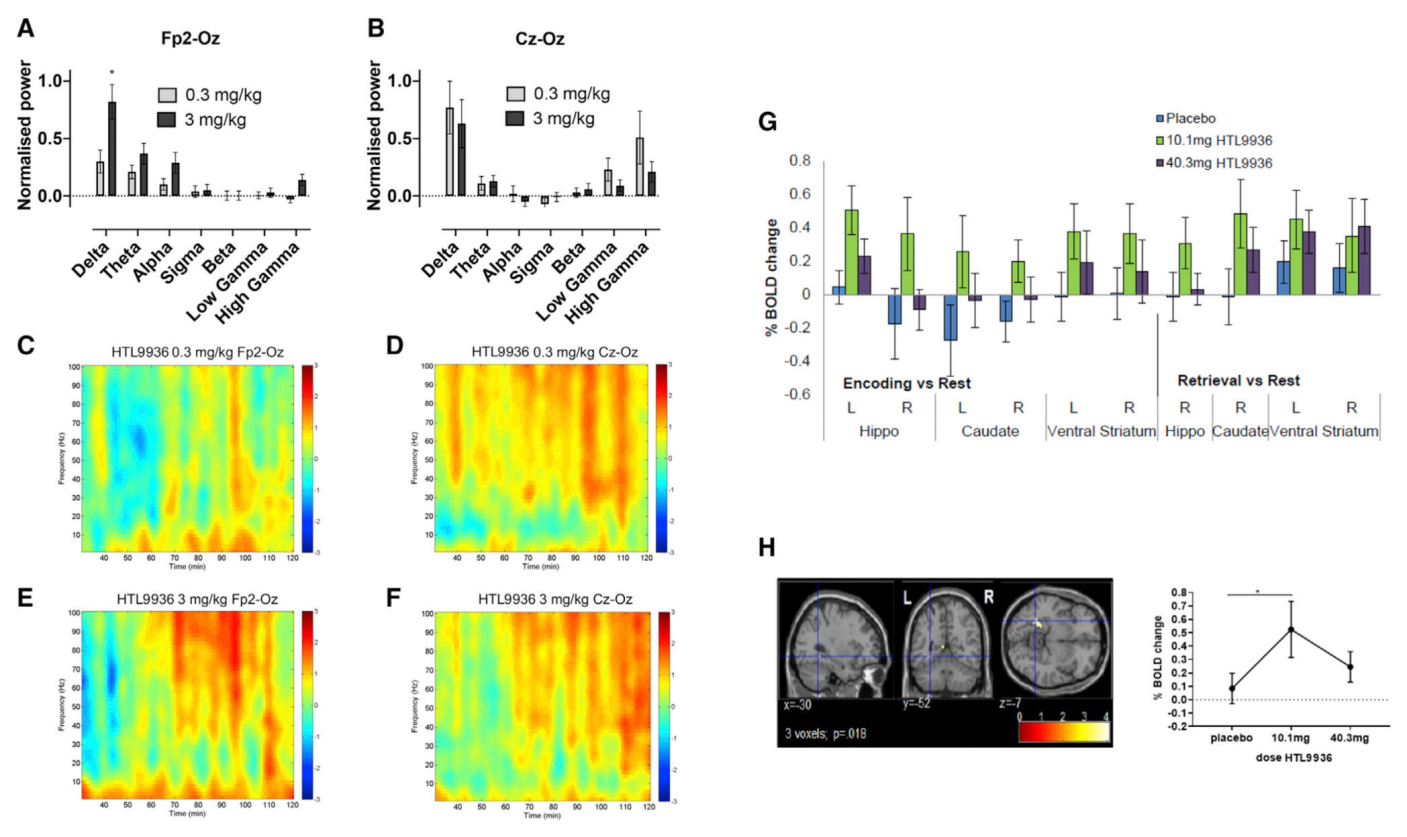
HTL9936 elicits robust changes in qEEG power spectra in cynomolgus monkeys and fMRI indicates target engagement in human volunteers (A and B) Dose-related changes in normalized power (to vehicle) across the Fp2-Oz (A) and Cz-Oz (B) electrode derivations. Results represent mean ± SEM of the AUC between 30 and 120 min after subcutaneous treatment with 0.3 and 1.0 mg/kg HTL9936 for 5 monkeys. *p < 0.05 versus vehicle-treated group by paired t test. (C−F) Time-frequency power spectrum showing resting state total power of the EEG at each frequency and time point, under HTL9936 normalized to time-by-time to vehicle treatment (t-values; frequency resolution—2 Hz; temporal resolution—1 min) for Fp2-Oz (C and E) and Cz-Oz (D and F) electrode derivations post-dose 30−120 min (90 min sampling window) for 0.3- (C and D) and 3.0 mg/kg (E and F) HTL9936 (N = 5). (G) Histogram illustrating fMRI in elderly human volunteers of the effects of HTL9936 on BOLD activation (expressed as a percentage signal change compared to rest) within regions associated with the Arena task (for the contrasts encoding versus rest and retrieval versus rest), (H) drug-induced signal change extracted from the left hippocampal activation during encoding (x = −30, y = −52, z = −7; Z_max_ > 3.8; p_svn_ = 0.018) plotted for each dose. Error bars represent the standard error of the mean. BOLD (Brain-oxygen-level-dependent); *p < 0.05 relative to placebo. See also Tables S5 and S6.

Rodent models are the mainstay of our attempts to understand mammalian brain function and as a tool for target validation and drug discovery. However, their ability to accurately predict human behavior depends on translational understanding of target function in the context of the behavior to be studied. Non-human primates offer an intermediate species in which to explore pharmacology between rodents and human. As a next step, we therefore examined the CNS exposure and preliminary evidence for CNS target engagement in non-human primates. We employed a basic quantitative EEG (qEEG) protocol to assess the effect of HTL9936 on resting state network firing in non-human primates. HTL9936 was dosed subcutaneously to avoid complications associated with the low oral bioavailability in primates (predicted brain exposures of Cu,br = 20 and 198 nM for 0.3 and 3 mg/kg, respectively) and resulted in increased delta power (Figures 7A−7F; Table S6). Delta oscillations have been linked with cognitive tasks (Basxar et al., 2001) where they are considered to suppress “non-relevant” neuronal activity necessary to allow for the execution of tasks such as working memory (Harmony, 2013).

### Evaluating the safety pharmacology of HTL9936—did the increased selectivity lead to an improved therapeutic margin in preclinical species?

Historically, the presence of classic cholinergic side effects, including salivation, sweating, convulsion/seizures, and gastrointestinal distress has resulted in dose-limiting toxicology and clinical adverse events thought to be mediated in part by the non-selective activation of M2- and M3-receptors (Conn et al., 2009b). As part of the safety evaluation necessary to support clinical development, HTL9936 was evaluated across a battery of CNS and cardiovascular (CV) safety studies.

Substantial literature highlights a potential role for the M1-receptor in proconvulsive/seizure-like behaviors (Bradley et al., 2020; Davoren et al., 2017; Hamilton et al., 1997; Rook et al., 2017). In a modified Irwin Functional Observation Battery test used to assess potential behavioral adverse effects in rats, there were no observations of significance adverse responses, including a complete absence of seizure-like effects on oral dosing up to 100 mg/kg (Cmax = 6,400 ng/mL; Cu,pl = 11,994 nM). In total across the entire HTL9936 program, there was only one convulsive-like episode recorded following treatment with HTL9936 in a 4-day repeat dose tolerability assessment in dogs. In this animal, the plasma Cmax was >32,000 ng/mL (Cu,pl = 59,971 nM), significantly above the exposures predicted to be required for cognitive improvements.

The cardiovascular effects of HTL9936 were initially tested in both telemetered rats and dogs. In rats, oral dosing of HTL9936 at 3, 10, 30, and 100 mg/kg resulted in a dose-dependent increase in heart rate and mean arterial blood pressure averaged over the 5 h post-dose period. No significant effects were evident at the 3 mg/kg dose. The effects of HTL9936 at all doses were transient in nature returning to baseline as the predicted exposure of HTL9936 declined. A similar dose-dependent profile of HTL9936 effects on blood pressure, but not heart rate, was seen in the dog GLP-compliant cardiovascular safety pharmacology telemetry study. All hemodynamic changes returned to control levels within 30 min of the end of the i.v. infusion (Table S6).

Consistent with other recently published studies on M1-receptor ligands (Alt et al., 2016; Davoren et al., 2016), HTL9936 was generally poorly tolerated in dogs compared with similar plasma exposures achieved in rats. Clinical signs attributable to M1-receptor agonism such as excessive salivation, vomiting, and lacrimation were observed in safety pharmacology and toxicology studies where the plasma exposure exceeded 157 ng/mL (Cu,pl = 283 nM). These observations were not evident in either rats or non-human primates with plasma exposure up to 732 and 557 ng/mL, respectively (Cu,pl = 1,372 and 991 nM, respectively), suggesting potential differences in species sensitivity to cholinergic adverse events.

In summary, HTL9936 demonstrated significant benefits across a range of established and novel models used to assess cognition with limited evidence for cholinergic AEs that have been associated with some historical programs. In order to help contextualise the safety, pharmacology, and efficacy data, the apparent therapeutic index (TI) was calculated by comparing the total plasma exposures between efficacy studies and the safety pharmacology/toxicology studies. These data are summarized in Table S6 and provide evidence of a therapeutic window that may be explored further in human studies.

### The profile of HTL9936 in healthy human volunteers

HTL9936 was progressed into a randomized, double-blind, placebo-controlled, first-time-in-human (FTIH) ascending single and multiple oral dose study in healthy young and elderly volunteers (ClinicalTrials.gov Identifier: NCT02291783). The single ascending dose (SAD) pharmacokinetics are summarized in Table S6.

The concentration of CNS drugs in the CSF have been widely used as a surrogate for the unbound drug concentrations in the brain in order to assess the potential for drugs to access their pharmacological targets. CSF drug levels were measured in 6 healthy male subjects after a single 54 mg oral dose of HTL9936. The maximum HTL9936 concentration in CSF was reached between 2−3 h, which was delayed relative to the plasma t_max_ (approximately 0.5 h). The mean ng/mL (±SD) ratio of CSF/plasma was 0.11 ± 0.04, 0.30 ± 0.05, and 0.39 ± 0.08 at 1, 2, and 3 h post-dose, respectively, with the rising ratio over time due to the relative persistence of HTL9936 in CSF compared with plasma. Based on the AUC_0-3h_, the mean (±SD) CSF/plasma ratio was 21 ± 3.4%. These data are consistent with pre-clinical CNS distribution data where CSF levels were measured.

In the SAD study, HTL9936 between 1−100mg showed no serious adverse events that led to withdrawal (Table S6). Mild responses consistent with cholinergic mechanisms (salivation, sweating, and changes in blood pressure and/or heart rate) were recorded only in the 175 mg cohort (3/5 subjects)(Table S6). Only subjects with a Cmax of 242 ng/mL recorded evidence of cholinergic side effects, which increased in duration and severity with increasing exposure (Table S6), In the context of therapeutic margins, the plasma drug concentrations of 242 ng/mL is about 5 × higher than the plasma minimal effective concentration in the aged beagle DNMP efficacy study (0.3 mg/kg dosed subcutaneously, Cmax plasma exposure 58 ng/mL). Based on the measured CSF to plasma ratios of HTL9936 from 1 to 3 h post-dose (11, 30, and 39, respectively), the plasma Cmax of 242 ng/mL can be estimated to represent unbound brain exposures in the range of 72 to 259 nM.

### CNS signal finding studies in human subjects

To understand whether HTL9936 would have sufficient safety margins between peripheral adverse cholinergic effects and CNS activity associated with cognition, it was helpful to identify a biomarker of CNS activity. Quantitative electroencephalogram (EEG) and Event Related Potentials (ERP) were included in the multiple dosing part of the study on day 1, day 4, and day 9 when HTL9936 would be predicted to have reached steady state. In a P300 active auditory oddball EEG paradigm, effects were found along frontal-parietal regions involved in generating attentional response and processing of stimuli. The high exposure group showed significant within- and between-group effects for deviant amplitude for electrodes Fz and Cz on day 9, with consistent but smaller effects on day 4. The deviant-standard results were consistent with the amplitude effects (Table S6).

The pharmacodynamic effects of HTL9936 on hippocampal dependent spatial learning and memory and hippocampal activity were examined in a separate study in elderly subjects (aged 65−80 years; ClinicalTrials.gov Identifier: NCT02546310). The study was a single-dose, double-blind, placebo-controlled functional magnetic resonance imaging (fMRI) study in which 54 healthy elderly subjects were randomly allocated to either placebo (0.9% sodium chloride aq. i.v.; n = 18) or HTL9936 (i.v.; n = 36) treatment. HTL9936 was administered via i.v. infusion in order to ensure control of HTL9936 exposure at steady-state within closely defined limits during the fMRI assessment. Two doses of HTL9936 were employed in this study with the aim of safely exploring preliminary evidence that the drug was active within CNS regions relevant to cognition. A low dose of 10.1 mg i.v. (n = 18) predicted to achieve steady-state plasma concentrations of 25 ng/mL and a high dose of 40.3 mg i.v. predicted to achieve steady-state plasma concentration of 100 ng/mL. Based on the measured human CSF to plasma ratios of HTL9936 these plasma exposures were predicted to range from approximately 7−30 nM and 30−110 nM for the low and high doses respectively (0.2- to 3.4-fold the human M1-receptor *in vitro* potency). Learning- and memory-related hippocampal activity was assessed using the Arena task, which is extensively described elsewhere (Antonova et al., 2009, 2011). Not unexpectedly, HTL9936 did not significantly improve task performance in this small number of healthy elderly subjects but did result in a significant drug by activation interaction effect in the left hippocampus during encoding of spatial cues, driven by increased activation under the 10.1 mg/kg dose, with the 40.3 mg/kg dose showing a smaller effect (F(1,30) = 6.44, p = 0.017 small volume corrected; Figures 7G and 7H). The 10.1 mg/kg dose consistently showed increased bilateral activation across the a-priori defined regions of interest during both encoding and retrieval phases of the task (Figures 7G and 7H). Taken together, these studies provide evidence of CNS target engagement for HTL9936 at concentrations with a safety profile suitable for further evaluation.

## Discussion

The publication of the first non-opsin GPCR crystal structures (Cherezov et al., 2007; Rasmussen et al., 2007; Rosenbaum et al., 2007) heralded the prospect that SBDD would unlock the full therapeutic potential of “hard-to-target” GPCRs such as the M1-receptor. Here we present the rational SBDD of a selective GPCR ligand with the expected *in vitro* and *in vivo* pharmacology that delivers preclinical efficacy in animal models and desirable animal and human toxicity that when tested in humans gave clinically relevant effects. In this way, we present a general road-map for the application of SBDD in GPCR-drug discovery. Specifically, our study demonstrates that it is possible to design a M1-receptor agonist (HTL9936) with positive effects on memory centers in humans at doses that show a significant reduction in the adverse effects that have thwarted previous attempts to target this receptor in AD.

To understand M1-receptor pharmacology at the atomic level, we generated multiple agonist-bound structures of the M1-receptor. Comparison of the binding of the extended orthosteric agonists 77-LH-28-1, GSK1034702, and HTL9936 provided support for a common proximal trigger of activation, involving disruption of the “tyrosine cage” seen previously in muscarinic-receptor antagonist structures (Thal et al., 2016). An appreciation of this allowed for the design of the size and orientation of the compound in this key activation region that acted to prevent the inward movement of Y104^3.33^, Y403^6.51^, and Y426^7.39^ seen in the M2-receptor iperoxo agonist structure (Fish et al., 2017). Appreciation of this resulted in the generation of compounds including HTL9936 with greatly reduced M2- and M3-receptor agonist activity but maintained nM activity at the M1-receptor.

Eliminating M2- and M3-receptor activity provided a clear rationale for the favorable safety profile of HTL9936 relative to orthosteric agonists such as xanomeline (Bender et al., 2017) and GSK1034702 (Nathan et al., 2013) that both show M2- and M3-receptor activity as well as clear cholinergic adverse responses in the clinic. There may, however, be other factors in play that contribute to the safety profile of HTL9936. Our data, and that of others, have established that both peripheral adverse responses and centrally mediated seizure and convulsions can be mediated by previously unappreciated on-target activity at M1-receptors (Bradley et al., 2020; Davoren et al., 2017; Engers et al., 2018). Here, our *in vitro* pharmacological characterization of HTL9936 revealed an un-biased signaling profile that, based on our previous studies (Bradley et al., 2020), would be predictive of a ligand with cognitive efficacy and minimal central and peripheral M1-related adverse effects.

Furthermore, an important feature of HTL9936 pharmacology is the partial agonist profile that likely contributes to low adverse events at clinically relevant concentrations. Previous pre-clinical studies have highlighted that centrally expressed M1-receptors exhibit a high level of receptor reserve (Porter et al., 2002). This suggests that M1-partial agonists such as HTL9936 can show sufficient efficacy to stimulate the well-coupled post-synaptic M1-receptors in the CNS while the partial nature of agonism means that peripheral M1-receptors expressed at lower levels are poorly activated. Importantly, the comparative analysis of M1-receptor crystal structures bound to HTL9936, and the full agonists 77-LH-28-1 and GSK1034702 provided a hypothesis for the molecular nature of partial agonism of HTL9936 that will aid future SBDD.

It should be noted that alternative approaches to those described here are currently being pursued to overcome the challenge of cholinergic-adverse that include xanomeline administered in combination with a peripheral non-selective muscarinic antagonist trospium (Brannan et al., 2021) and M1-receptor specific positive allosteric modulators that potentiate the action of endogenous acetylcholine (Conn et al., 2009b; Voss et al., 2018; Moran et al., 2018).

Importantly for the design of future clinical trials using HTL9936, or related molecules, is the observation reported here that HTL9936 has efficacy both alone and in combination with donepezil indicating that combination trails with current standards of care should be feasible.

In conclusion, we describe the rational design of a partial M1-agonist, HTL9936, that delivers a therapeutic window between desirable pro-cognitive activity and peripheral cholinergic adverse effects. In this way, we not only provide the basis for the development of effective drugs for the treatment of AD but also describe a general road-map for the application of SBDD in GPCR-drug discovery that realizes the promise of rational ligand design made over a decade ago by the report of the first GPCR structures.

## Star★Methods

Detailed methods are provided in the online version of this paper and include the following:

- KEY RESOURCES TABLE
- RESOURCE AVAILABILITY
  ∘ Lead contact
  ∘ Materials availability
  ∘ Data and code availability
- EXPERIMENTAL MODEL AND SUBJECT DETAILS
  ∘ Cell Culture
  ∘ Use of pre-clinical animal species
  ∘ Rat intravenous infusion in non-GLP cardiovascular study
  ∘ Dog intravenous infusion in GLP cardiovascular study
  ∘ Beagle aged studies
  ∘ Non-human primate studies
  ∘ Toxicology studies on HTL9936
  ∘ Human experimental subjects
- METHOD DETAILS
  ∘ GPCR residue numbering
  ∘ Primary neuronal culture preparations
  ∘ Drug discovery screening strategy
  ∘ Generation of the M1-receptor StaR variant
  ∘ StaR radioligand binding assay
  ∘ Thermostability measurement of M1-receptor variants
  ∘ Expression, membrane preparation and protein purification of M1-receptor variants
  ∘ Procedures for synthesis of HTL9936
  ∘ Steps for the synthesis of HTL9936 are as follows
  ∘ Summary of synthesis of HTL9936
  ∘ IP1 accumulation assay performed on CHO-FlpIn cells expressing the M1-receptor
  ∘ IP1 accumulation assay performed on HEK293T cells expressing the M1-receptor
  ∘ [^3^H]-NMS binding to the M1-receptor
  ∘ pERK1/2 assay on CHO-K1 cells expressing muscarinic receptors
  ∘ pERK assay on CHO-K1 cells expressing muscarinic receptors
  ∘ Arrestin interaction assay with the M1-receptor
  ∘ Determination of the internalisation of the M1-receptor
  ∘ Activation of G-proteins G_q_/G_11_/G_15_
  ∘ GTPγS assay to establish G protein activation by the M1-receptor
  ∘ Pharmacokinetic analyses of HTL9936 in animals undergoing fear conditioning
  ∘ Novel object recognition behavioral test
  ∘ Passive avoidance test behavioral test
  ∘ Open field behavior behavioral test
  ∘ Fear conditioning behavioral test
  ∘ Prion-infection of mice
  ∘ Delayed non-matching to position task (DNMP)
  ∘ Quantitative EEG (qEEG) resting state in macaques
  ∘ Rat cardiovascular study of the effects of HTL9936 administration
  ∘ Dog intravenous infusion GLP cardiovascular study
  ∘ Clinical drug administration
  ∘ Clinical EEG recordings
  ∘ Clinical functional MRI
- QUANTIFICATION AND STATISTICAL ANALYSES
  ∘ Radioligand Binding
  ∘ Crystallography Data processing
  ∘ Structure solution and refinement
  ∘ Molecular dynamics simulations
  ∘ Extracellular cavity volume analysis
  ∘ Bias factor calculations
  ∘ Dog intravenous infusion GLP cardiovascular study
  ∘ Non-human primate qEEG measurments
  ∘ Clinical EEG measurements
  ∘ Clinical functional MRI

## Star ★Methods

### Key Resources Table

**Table:** 

REAGENT or RESOURCE	SOURCE	IDENTIFIER
Chemicals, peptides, and recombinant proteins		
GF/B filters	Merck	WHA1821047
UltraGold Scintillation	Perkin Elmer	6013329
Complete protease inhibitor cocktail	Merck	11697498001
Superflow resin	QIAGEN	30410
Superdex 200	Sigma-Aldrich	GE28-9909-44
Atropine	Sigma-Aldrich	A0132-1G
GSK1034702	In house synthesis	N/A
Acetylcholine (chloride)	Sigma-Aldrich	A6625-10mgs
Donepezil	Tocris	4385
[3H]-77-LH-28-1	RC Tritec, Switzerland	Custom radionucleotide synthesis 48.01Ci/mmol
[3H]-NMS	PerkinElmer	NET636250UC
DMEM (high glucose w-out sodium pyruvate)	Invitrogen	41965-039
FBS (heat inactivated)	Invitrogen	10500064
GeneJuice	Sigma-Aldrich	70967
Poly-d-lysine coated plates	VWR	734-0120
Hanks Balanced salt solution (HBSS)	Merck	H4641
ESF 921 medium	Expression systems	96-001-01
384 well white proxiplates	Cole Parmer (UK)	781358
Critical commercial assays		
Alphascreen Surefire pERK assay	Perkin Elmer	ALSU-PERK-A-HV
IP-One Tb assay kit	CisBio	621PAPEC
Bac to Bac Expression System	ThermoFisher	10359016
Deposited data		
M1-StaR-T4L in complex with 77-LH-28-1	This paper	6ZFZ
M1-StaR-T4L in complex with HTL9936	This paper	6ZG4
M1-StaR-T4L in complex with GSK1034702	This paper	6ZG9
β_1_-adrenergic receptor:cyanopindolol	Warne et al., 2008	2VT4
Phase1 Healthy Volunteer SAD/MAD	Clinicaltrials.gov	NCT02291783
Cognition and BOLD fMRI Healthy Elderly Subjects	Clinicaltrials.gov	NCT02546310
Phase1 Healthy Volunteer SAD/MAD	EUDRACT.ema.europa.eu	2013-002307-34
Cognition and BOLD fMRI Healthy Elderly Subjects	EUDRACT.ema.europa.eu	2015-002490-38
Experimental models: Cell lines		
HEK293T	Sigma-Aldrich	12022001-Cdna-20ul
CHO-FlpIn	ThermoFisher	R75807
Experimental models: Organisms/strains		
Mice C57BL/6 (male aged 8-12weeks)	Charles River	N/A
Rats (adult male Hans Wistar age 11weeks)	Harlan, UK	N/A
Beagle dogs (male and female 32to 48weeks)	Harlan, UK	N/A
Cynomolgus macaques (Mascara fasicularis) male	SRI, US	N/A
Mice C57BL/6 (male aged 8-12weeks)	Charles River	N/A
Mice Tg37 (male homozygous mice)	Mallucci et al., 2003; 5646 pp871	N/A	
Software and algorithms		
Schrodinger Glide softwear	Schrodinger LLC	https://www.schrodinger.com
Ethivision XT,	Noldus UK	https://www.noldus.com/
DataQuest ART	DSI, St Paul, MN	Dataquest ART https://www.datasci.com/products/software/dataquest-art
Phaser	McCoy et al., 2007	https://www.phaser.cimr.cam.ac.uk/index.php/Downloads
MolProbity	Chen et al., 2010	http://molprobity.biochem.duke.edu/
POINTLESS	Winn et. al. 2011	https://www.ccp4.ac.uk/
AIMLESS	Evans and Murshudov 2013	https://www.ccp4.ac.uk/
MOSFLM	Battye et al. 2011	https://www.mrc-lmb.cam.ac.uk/mosflm/mosflm/
XDS	Kabsch, 2010	https://xds.mr.mpg.de/
COOT	Emsley et al., 2010	https://www.mrc-lmb.cam.ac.uk/personal/pemsley/coot/
MATLAB	The MathWorks, Inc., USA	https://www.mathworks.com
Brainvision Analyzer software	Brain Products Gilching, Germany	https://brainvision.com
ImageJ	Noldus UK	https://imagej.nih.gov
BUSTER	Globalphasing Ltd	https://www.globalphasing.com
PyMOL	Schrodinger LLC	https://pymol.org/2/

### Resource Availability

#### Lead contact

Further information and requests for resources and reagents should be directed to and will be fulfilled by the lead contact,. Andrew Tobin (andrew.tobin@glasgow.ac.uk)

## Experimental Model and Subject Details

### Cell Culture

HEK293T and Chinese hamster ovary (CHO-K1) cells stably expressing recombinant M1-receptors were maintained in Dulbecco’s Modified Eagle’s Medium (DMEM) supplemented with 0.292 g.l^-1^ L-glutamine, penicillin/streptomycin mixture and 10% (v/v) fetal bovine serum (FBS) at 37°C in a 5% CO_2_ humidified atmosphere. For experiments using transiently transfected HEK293T cells, transfections were carried out using 1 mg/mL polyethyleneimine (PEI) (MW-25000) and experiments conducted 48 h post transfection. FlpInCHO cells (Thermo Fisher Scientific) transfected to stably express recombinant human M1-receptor were grown in T75cm^2^ flasks in Ham’s F-12 media containing 10% fetal bovine serum and 1% penicillin/streptomycin and under hygromycin B selection (400 mg/mL) were induced to express the M1-receptor by treatment with 100 ng.ml^-1^ doxycycline for 24 h.

### Use of pre-clinical animal species

Mouse work reported here was conducted on male and female C57/B6 mice or female Tg37 transgenic line overexpressing mouse prion protein (Mallucci et al., 2003) under the UK Home Office Project license PPL7008473, PP7704105 and PP7704105. Experimentally naive male Wistar rats were purpose bred at Harlan, UK. Animals were introduced to the experimental holding rooms 5 days prior to the commencement of the study, housed in groups of 4 during this period, and maintained at 22-24°C on a standard 12 hour-light/dark cycle, with food and water available *ad libitum*. All animals were examined and weighed daily. Rat *in vivo* studies were approved by University College Dublin ethics committee and carried out by individuals licensed by the Irish Department of Health according to current European legislation (Directive 86/609EEC).

### Rat intravenous infusion in non-GLP cardiovascular study

The study was performed using 7 male Wistar rats (JANVIER LABS, C.S. 4105, Saint Berthevin F-53941 France), weighing 220-300 g on the day of surgical implantation. Animals were housed in groups of 2-4 in polysulfone cages (floor area = 1500 cm^2^) under standard conditions: room temperature (22 ± 2°C), light/dark cycle (12h/12h), air replacement (15-20 volumes/hour), water and food (SDS, RM1) *ad libitum*. Animals were allowed to habituate for at least 5 days prior to surgery.Following surgical procedures animals were individually housed. The study was conducted under EU and French animal welfare regulation for animal use in experimentation (European Directive 2010/63/EEC and French decree and orders of February 1st 2013). HTL9936 or vehicle were administered by gavage (4mL/kg) at time = 0. Summary profiles representing the mean of HTL9936 across 4-6 animals at 3, 10, 30 and 100 mg/kg on heart rate and mean arterial blood pressure are depicted below. Arterial blood pressure and heart rate (determined from the pulsed blood pressure signal) were continuously measured 60 min before administration of HTL9936 or vehicle. At each time point the results were averaged over the preceding 5 min period (Figure S7A,B).

The rat GLP cardiovascular study was conducted under EU and French animal welfare regulation for animal use in experimentation (European Directive 2010/63/EEC and French decree and orders of February 1^st^ 2013).

### Dog intravenous infusion in GLP cardiovascular study

Six male pure-bred beagle dogs were obtained from Harlan, UK. The animals were housed in an air-conditioned room to provide a minimum of 15 to 20 air

changes/hour. Routinely, the temperature was maintained within acceptable limits (nominally 15 to 21°C). On one occasion the temperature exceeded the protocol specification and was recorded at 26°C. Fluorescent lighting was controlled automatically to give a cycle of 12 hours light and 12 hours dark. Each animal was offered approximately 300 g of 5L66 Certified High Density Canine Diet (IPS Product Supplies Ltd., London) each morning. The study was designed to meet the requirements of ICH Guideline (Topic S7A;CPMP/ICH/539/00) on Safety Pharmacology Studies for Human Pharmaceuticals (November 2000). Animals were implanted with calibrated sensors (DSI D70 Series) for arterial blood pressure (ABP). The signals were processed by the Open Art/ PoNeMah data acquisition/analysis software. Vehicle and test article were administered by intravenous infusion over 2 hours, using a constant dose volume of 10 mL/kg (5 mL/kg/hr) and data was continuously recorded at logging rates of 1 min. Two pre-dose readings of the heart rate, blood pressure (Diastolic, systolic and mean arterial pressure) and ECG intervals (PR-, QRS-, QT- and QTCF) were taken 15 min apart before administration of each dose. Following the start of the infusion, of vehicle or test article, readings were taken from the line averages at 15, 30, 45, 60, 75, 90, 105 and 120 min (i.e during the infusion), 150 and 180 min (i.e., 0.5 and 1 hour post infusion in sling) (additional recordings were continued to 12hours post-dose). For each variable, the mean of the pre-dose values recorded for each animal on each dose were taken as the baseline for that dose and that animal. Each time point was analyzed using ANOVA, fitting DOSE and ANIMAL as fixed effects with the baseline value as a covariate. Pairwise comparisons of each dose with control (Dose 1) were made using Dunnett’s test (Figures S7C and S7D).

### Beagle aged studies

Adult male and female Beagle dogs were housed individually. A combination of commercially available artificial light and natural light was provided to the animals. Heating and cooling was electronically maintained and set to maintain the temperature in a range from 15°C to 28°C with the room ventilation designed to provide 18 filtered air changes per hour. All animal facilities were cleaned daily. The animals were fed Purine Pro Plan to maintain bodyweight at the end of each day with access to ffod after the study protocol on test occasions. Water was provided *ad lib*. Protocols for the aged beagle dog studies were approved by Vivocore Internal Animal Care and Use Committee (IACUC) in accordance with the Canadian Council on Animal Care (CCAC).

### Non-human primate studies

Adult male Cynomolgus macaques (Macaca fasicularis) were used for these studies. Subjects were house in a temperature controlled recording room maintained on a 12-hour light/dark cycle. They had access to water *ad libitum* and are fed a full daily regimen of food. In addition to their food,the animals are provided with fresh fruits and vegetables daily and their welfare supported with additional environmental enrichment strategies (access to toys, ambient sounds and visual stimulation).

All studies were conducted in compliance with USDA and SRI International IACUC guidelines. SRI International is an AAALAC accredited institution.

### Toxicology studies on HTL9936

The rat and dog were appropriate species in which to conduct nonclinical safety evaluation and HTL9936. Male and female rates of the RccHan: WIST strain were obtained from Harlan, Bicester, UK. and the animals were approximately seven to eight weeks old at the start of dosing. Throughout the study the animals had access *ad libitum* to SQC Rat and Mouse Maintenance Diet No 1, Expanded, (Special Diets Services Ltd, Witham, UK). Rooms were air-conditioned to provide 15 to 20 air changes/hour. The temperature and relative humidity ranges were maintained in the specified ranges of 20 to 24°C and 45 to 65%, respectively. Fluorescent lighting was controlled automatically to give a cycle of 12 hours light and 12 hours dark. The animals were given wooden Aspen chew blocks and rodent retreats that did not require analyses as forms of environmental enrichment.

Beagle dogs were aged between 32 to 40 weeks old at the start of dosing. Animals were offered 5L66 Certified High Density Canine Diet (IPS Product Supplies Ltd, London) each day and water was provided *ad libitum* via an automatic watering system. Rooms were air-conditioned to provide 15 to 20 air changes/hour and the temperature range was maintained in the specified range of 15 to 21°C. Fluorescent lighting was controlled automatically to give a cycle of 12 hours light and 12 hours dark. Environment enrichment included the provision of toys (such as balls, inert nylon chews), raised platforms and exercise/socialisation periods. Details were maintained in the study record

Toxicology studies were conducted males and female of both species under the UK Home Office Project License PPL60/3774 and where appropriate in accordance with the OECD Principle on Good Laboratory Practice ENV/MC/CHEM (98)17.

### Human experimental subjects

HTL9936 was progressed into a randomized, double blind, placebo controlled, first time-in-human (FTIH) ascending single and multiple oral dose study in healthy young adult male volunteers and healthy elderly male and female volunteers. (ClinicalTrials.gov Identifier: NCT02291783).

The pharmacodynamic effects of HTL9936 on hippocampal dependent spatial learning and memory and hippocampal activity were examined in a separate study in heqalthy elderly male and female subjects (aged 65-80 years; ClinicalTrials.gov Identifier: NCT02546310).

## Method Details

### GPCR residue numbering

The generic GPCR residue numbering system (Isberg et al., 2015) used throughout this paper is based on the based on the Ballesteros-Weinstein residue numbering system includes two numbers (X.N), the first (1-7) denotes the transmembrane helix (TM), and the following number indicates the residue position relative to the most conserved amino-acid in the helix (which is assigned the number 50). Conserved residue positions in Extracellular Loop 1 (EL1, between TM2 and TM3) and Extracellular Loop 2 (EL2, between TM4 and TM5) are defined as W23.50 and C45.50, respectively. For example, 3.33 indicates the residue 17 positions before the most conserved amino-acid in Class A GPCR TM3 (R3.50). If an amino acid is followed by its residue number, the generic GPCR residue numbering is included as superscript.

### Primary neuronal culture preparations

Tissue culture plates were coated using 4 μg.ml^-1^ poly-D-lysine and 6 μg.ml^-1^ Laminin Mouse Protein in DEPC treated H_2_O and incubated overnight at 37°C. Plates were then washed three times using DEPC treated H_2_O and dried for 2h at room temperature.

The hippocampal and cortical areas of the brain were isolated from E16 embryos. The tissues were chopped into smaller pieces, washed three times in Hanks’ balanced salt solution (HBSS), transferred to a 15 mL tube containing 4ml of TrypLE Select 10X and incubated at 37°C for 10 min. TrypLE Select 10X was then inactivated by adding 8 mL of neurobasal complete media (Neurobasal Plus medium supplemented with 20 ml.L^-1^ B-27 plus, 0.292 g.L^-1^ L-glutamine, 100 U.ml^−1^ penicillin, 0.1 mg.ml^−1^ streptomycin) to the tubes followed by centrifugation at 200 x g for 5 min. The pellet was resuspended in neuro-basal complete media to a final density of 5x10^5^ cells/mL^-1^. Cells were then seeded onto pre-coated plates and maintained at 37°C in a 5% CO_2_ humidified atmosphere.

### Drug discovery screening strategy

The protein preparation and docking experiments were done within the Schrödinger Maestro package. The grid generation necessary for docking was done within Glide. The residues highlighted in SDM experiments (in-house and external) were used to further define the cavity of the grid. However, no constraints were added in the grid generation to ensure that subsequent dockings were not biased in any way. As standard, up to 3 poses per molecular structure were stored for analysis.

1.6 million fragment-like compounds were prepared for screening, and all or a subset from more stringent prefiltering and clustering were docked into each of the models using the SP algorithm within the Schrödinger Glide software, running on a 28 CPU Linux cluster. The resultant hits from the virtual screen were then reduced further by applying distance and volume constraints to ensure that the bound poses were within the area highlighted by SDM to be important for agonism of the structure.

The more stringent subset was generated from a ligand-based scaffold hopping approach whereby common motifs within the known agonists, highlighted above, were used as a preselection filtering criterion to enrich the compound set for subsequent docking.

### Generation of the M1-receptor StaR variant

To alleviate toxicity associated with DNA propagation of WTM1-receptor, a hybrid construct comprising residues 1-95 of M4 fused to residues 88-438 of M1-receptor was used as the template for StaR generation which was found to minimize toxicity commonly associated with high expression levels while minimizing the number of amino acid substitutions. A single amino acid substitution Trp101^3.28^ (W101A) was introduced to increase the affinity for the M1-receptor agonist 77-LH-28-1. Conformational thermostabilization was performed using a mutagenesis approach previously described (Magnani et al., 2016; Robertson et al., 2011). Mutants were analyzed for thermostability in the presence of the radioligand [^3^H] 77-LH-28-1. The final StaR contained 12 thermostabilizing mutations (F27A^1.34^, T32A^1.39^, V46L^1.53^, L64A^2.43^, T95A, W101A^3.28^, S112A^3.39^, A143L^4.43^, A196T^5.46^, K362A^6.32^, A364L^6.34^, S411A^7.46^ − note these 12 mutations are referred to as γ12.1 in the constructs described below).

### StaR radioligand binding assay

Radioligand binding using [3H]-77-LH-28-1 was performed on HEK293T membranes following 48 h transient transfection of M1/M4 W101A, M1/M4 W101A γ12.1 StaR or M1/M4 W101A γ12.1 T4L StaR. For confirmation of receptor expression to support functional characterization in the pERK assay, binding was also performed on CHO-K1 membranes stably expressing M1/M4 W101A γ12.1 StaR. All experiments were performed in 96 well format over 2 h at room temperature using 5ug/well protein in a final volume of 400ul of assay buffer of the following composition: 20 mM HEPES, 100 mM NaCl and 10 mM MgCl2, pH 7.4 Binding reactions were terminated by rapid filtration through GF/B filters (Perkin Elmer, Boston, MA, USA) pre-soaked with 0.5% w/v PEI for 1 h. Filters were then washed 3 times with 1 mL ice-cold assay buffer. Dried filters were counted with UltimaGold scintillant (Perkin Elmer) using a Microbeta (Perkin Elmer, Boston, MA, USA). The specific bound counts (d.p.m.) were expressed as a percentage of the maximal binding observed in the absence of test compound (total) and non-specific binding determined in the presence of 10 μM atropine.

Affinity (K_D_) for [^3^H] 77-LH-28-1 for each construct was determined in membranes by saturation binding assays, performed by incubating increasing concentrations of [^3^H] 77-LH-28-1 in the absence or presence of 10μM atropine. Radioligand inhibition binding assays were performed by co-incubating membranes with increasing concentrations of test compounds and an approximate 5x concentration of the equilibrium dissociation constant (K_D_) concentration of [^3^H] 77-LH-28-1 (K_D_ for [^3^H] −77-LH-28-1 binding to HEK293T cell membranes expressing the M1/M4 W108A γ 12.1 StaR was 0.06 ± 0.03 nM; n = 3)

### Thermostability measurement of M1-receptor variants

Transiently transfected HEK293T cells were incubated in 50mM sodium citrate pH 6.4, 150mM NaCl, 200nM [^3^H] 77-LH-28-1 supplemented with Complete Protease Inhibitor Cocktail tablet (Roche) for 2 h at room temperature. All subsequent steps were performed at 4°C. Cells were solubilised in 2% (w/v) *n*-nonyl-β-D-glucopyranoside (NG), for 1 h and crude lysates clarified by centrifugation at 16,000 g for 15 min. Receptor thermostability was measured by incubation at varying temperatures for 30 min followed by separation of unbound radioligand by gel filtration. Levels of ligand-bound receptor were determined using a liquid scintillation counter. Thermal stability (Tm) is defined as the temperature at which 50% ligand binding is retained.

### Expression, membrane preparation and protein purification of M1-receptor variants

To facilitate crystallization further modifications were made to the M1-StaR construct. The flexible domains were removed from the N terminus (residues 1-27) and C terminus (residues 439-460) and T4-lysozyme (T4L) was inserted into intracellular loop 3 (ICL3) between residues 219 and 354. These modifications did not alter the ligand binding properties of the receptor compared to wild-type M1-receptor. The construct further comprises an N-terminal GP64 signal sequence, and a C-terminal deca-histidine tag.

The receptor was expressed using the Bac to Bac Expression System (Invitrogen) in *Spodoptera frugiperda* Sf21 cells using ESF 921 medium (Expression Systems) supplemented with 10% (v/v) fetal bovine serum (Sigma-Aldrich) and 1% (v/v) Penicillin/Streptomycin (PAA Laboratories). Cells were infected at a density of 3.5 ×10^6^ cells/mL with virus at an approximate multiplicity of infection of 2. Cultures were grown at 27°C with constant shaking and harvested by centrifugation 48 hours post infection. All subsequent protein purification steps were carried out at 4°C unless otherwise stated.

For each protein preparation, cells from 5L cultures were resuspended in phosphate buffer saline (PBS) supplemented by 5 mM EDTA and Complete EDTA-free protease inhibitor cocktail tablets (Roche). Cells were disrupted at ~15 000 psi using a microfluidizer (Processor M-110L Pneumatic, Microfluidics). Membranes pelleted by ultra-centrifugation at 200 000 g for 50 min, were subjected to two successive high salt washes in a buffer containing PBS, 1 M NaCl and Complete EDTA-free protease inhibitor cocktail tablets, before they were centrifuged at 200,000 g for 50 min. Washed membranes were resuspended in 125 mL 40 mM Tris pH 7.6 and 500 mM NaCl supplemented with Complete EDTA-free protease inhibitor cocktail tablets and stored at −80°C until further use.

Membranes were thawed, resuspended in a total volume of 150 mL with 40 mM Tris−HCl pH 7.6, 500 mM NaCl, Complete EDTA-free protease inhibitor cocktail tablets (Roche), 40 μM HTL9936 (or 40 μM GSK1034702) and incubated for 40 min at room temperature. Membranes were then solubilized by addition of 1.5% (w/v) n-Dodecyl-β-D-maltopyranoside (DDM, Anatrace), and incubation for 1 hours at 4°C, followed by centrifugation at 145 000 g for 60 min to harvest solubilised material.

The solubilised material was batch bound for 3 hours to Ni-NTA (nickel-nitrilotriacetic acid) Superflow resin (QIAGEN) pre-equilibrated in 40 mM Tris-HCl pH 7.6, 150 mM NaCl, 10 mM imidazole, 0.05% DDM, 20 μM HTL9936 (or 20 μM GSK1034702 or 77-LH-28-1). the resin was then packed in a cartridge, and the column was washed with a gradient of 10 to 60 mM imidazole over 35 column volumes in a buffer containing 40 mM Tris-HCl pH 7.6, 500 mM NaCl, 0.05% (w/v) DDM, 70 mM imidazole, 20 μM HTL9936 (or 20 μM GSK1034702 or 77-LH-28-1) and then the protein was eluted with 40 mM Tris-HCl pH 7.6, 500 mM NaCl, 0.05% DDM, 245 mM imidazole, 20 μM HTL9936 (or 20 μM GSK1034702 or 77-LH-28-1). The receptor was further purified by size exclusion chromatography using a Superdex 200 column (GE Healthcare) pre-equilibrated with 40 mM Tris-HCl pH 7.6, 150 mM NaCl, 0.03% (w/v) DDM and 40 μM HTL9936 (or 40 μM GSK1034702 or 20 μM HTL9936). Receptor purity was assayed using SDS-PAGE and LC-MS and receptor monodispersity was assayed using analytical SEC. The protein was typically concentrated to ~60 mg/mL using a 100 KDa cut off membrane prior to crystallization setups.

### Procedures for synthesis of HTL9936

Where no preparative routes are included, the relevant intermediate was commercially sourced. Commercial reagents were utilized without further purification. Room temperature (rt) refers to approximately 20-27°C. ^1^H NMR spectra were recorded at 400 MHz on either a Bruker or Jeol instrument. Chemical shift values are expressed in parts per million (ppm), i.e., (5)-values. The following abbreviations are used for the multiplicity of the NMR signals: s = singlet, br = broad, d = doublet, t = triplet, q = quartet, quint = quintet, td = triplet of doublets, tt = triplet of triplets, qd = quartet of doublets, ddd = doublet of doublet of doublets, ddt = doublet of doublet of triplets, m = multiplet. Coupling constants are listed as J values, measured in Hz. NMR and mass spectroscopy results were corrected to account for background peaks. Chromatography refers to column chromatography performed using 60-120 mesh silica gel and executed under nitrogen pressure (flash chromatography) conditions. TLC for monitoring reactions refers to TLC run using the specified mobile phase and the silica gel F254 as a stationary phase from Merck. Microwave-mediated reactions were performed in Biotage Initiator or CEM Discover microwave reactors. Mass spectroscopy was carried out on Shimadzu LC-2010 EV, Waters ZQ-2000, UPLC-Mass SQD-3100 or Applied Biosystem API-2000 spectrometers using electrospray conditions as specified for each compound in the detailed experimental section. Preparative HPLC was typically carried out under the following conditions, (Waters HPLC): Column: XSelect CSH Prep C-18, 19 × 50 mm, 5μηι; Mobile phase: Gradients of water and MeCN (each containing 0.1% Formic Acid); gradient 5% MeCN in 0.1 HCOOH in water (30 s), 5% to 40% (over 7 min) then 95% MeCN in 0.1 HCOOH in water (1 min) then 5% MeCN in 0.1 HCOOH in water (1.5 min) at 28 mL / min.

LCMS experiments were typically carried out using electrospray conditions as specified for each compound under the following conditions;

***Method A and B:*** Instruments: Waters Alliance 2795, Waters 2996 PDA detector, Micromass ZQ; Column: Waters X-Bridge C-18, 2.5 micron, 2.1 ×20 mm or Phenomenex Gemini-NX C-18, 3 micron, 2.0 ×30 mm; radient [time (min)/solvent D in C (%)]: **Method A**: 0.00/2, 0.10/2, 2.50/95, 3.50/95, 3.55/2, 4.00/2 or **Method B**: 0.00/2, 0.10/2, 8.40/95, 9.40/95, 9.50/2, 10.00/2; Solvents: solvent C = 2.5 L H_2_0 + 2.5 ml_ ammonia solution; solvent D = 2.5L MeCN + 135 mL H_2_0 + 2.5 ml_ ammonia solution); Injection volume 3 μl; UV detection 230 to 400nM; column temperature 45°C; Flow rate 1.5 mL/min.

***Method C:*** Instrument: LCMS (Agilent 1200-61 10) with UV and ELSD detector at 40°C using waters X-Bridge C18 (4.6 mm*50 mm, 3.5 μm) and using water (0.05% TFA) and acetonitrile (0.05% TFA) as the mobile phase. The eluent gradient program was MECN (0.05% TFA) from 5% to 100% for 1.6 min and 100% MECN (0.05% TFA) for 1.4 min. The flow rate was 2.0 mL/min.

***Method D:*** Instrument: LCMS (Agilent 1200-61 10) with UV and ELSD detector at 40°C using waters X-Bridge C18 (4.6 mm*50 mm, 3.5 μm) and using water (0.05% TFA) and acetonitrile (0.05% TFA) as the mobile phase. The eluent gradient program was MECN (0.05%TFA) from 5% to 100% for 5 min and 100% MeCN (0.05% TFA) for 1.0 min. The flow rate was 2.0 mL/min.

### Steps for the synthesis of HTL9936 are as follows

**STEP 1**. A mixture of 4-oxoazepane-1-carboxylic acid *tert*-butyl ester (90 g, 422 mmol) and (R)-1-phenylethanamine (56.4 g, 465 mmol) in THF (1000 ml) was stirred at rt for 15 min and STAB (107.4 g, 510 mmol) was added. The mixture was cooled to 0°C in an ice bath, then acetic acid (26.7 g, 450 mmol) was added. The mixture was stirred overnight at rt then concentrated *in vacuo*, residue dissolved in DCM (800 ml) and washed with sat. NaHCO_3_ sol. (2 × 300 ml), dried (Na_2_SO_4_). The solvents were removed *in vacuo*, and the residue was purified by column chromatography (gradient 0% to 3% MeOH in DCM) to give tert-butyl 4-{[(1 R)-1-phenylethyl]amino}azepane-1-carboxylate (90 g, 67.0%) as a mixture of two diastereoisomers. 70 g of this mixture was separated by chiral prep. HPLC [Instrument: Waters Thar-SFC 200 with UV detector GILSON UV-1 (−151/152/155/156) at 35°C using CHIRALPAK AY-H (2.0 cm I.D. x 25 cm L. 5 μm) and using (Acetonitrile/iso-propanol)(0.2%DEA)/CO2 = 1.2/4.8/94 (V/VV) as the mobile phase. Flow rate was 120 mL/min (all solvents were HPLC grade). The system back pressure was 100bar. The SFC system was monitored at 214 nm.] to afford *tert*-butyl (4S)-4-{[(1 R)-1-phenylethyl]amino}azepane-1-carboxylate (26 g, 24.9% yield) as a yellow oil 1H NMR: (400MHz, CDCI3) δ: 1.26 (d, J = 7.1, 3H), 1.33 (s, 9H), 1.34 - 1.43 (m, 3H), 1.72 - 1.97 (m, 3H), 2.34 - 2.39 (m, 1 H), 3.01 - 3.45 (m, 4H), 3.80 (q, J = 7.2, 1 H), 7.15 - 7.25 (m, 5H), NH proton not observed. [a]D^20^ = + 57.0 (c = 0.5 in MeOH) and *tert*-butyl (4R)-4-{[(1 R)-1-phenylethyl]amino}azepane-1-carboxylate (30 g, 28.6% yield) as a yellow oil. 1H NMR: (400MHz, CDCI3) δ: 1.27 (d, J = 7.0, 3H), 1.34 (s, 9H), 1.34 - 1.42 (m, 3H), 1.74 - 1.96 (m, 3H), 2.35 - 2.41 (m, 1 H), 3.02 - 3.45 (m, 4H), 3.81 (q, J = 7.1, 1 H), 7.16 - 7.26 (m, 5H), NH proton not observed. [a]D^20^ = - 31.8 (c = 0.5 in MeOH)

The absolute configuration of *tert*-butyl (4S)-4-{[(1 R)-1-phenylethyl]amino}azepane-1-carboxylate was determined by X-ray analysis of the p-bromobenzoate salt of tert-butyl (4S)-4-{[(1 R)-1-phenylethyl]amino}azepane-1-carboxylate.

**STEP 2**. A suspension of Pd(OH)_2_/C (10%, 550 mg), *tert*-butyl (4S)-4-{[(1 R)-1-phenylethyl]amino}azepane-1-carboxylate (5.5 g, 17.3 mmol) and HCOONH_4_ (3.3 g, 51.9 mmol) in MeOH (80 ml) was heated at reflux for 1.5 h. The reaction mixture was cooled to rt and filtered; the solvents of the filtrate were removed *in vacuo*. The residue was purified by column chromatography (gradient 0% to 10% MeOH in DCM) to give *tert*-butyl (4S)-4-aminoazepane-1-carboxylate (3.2 g, 87.3%). 1H NMR: (400MHz, CDCI3) δ: 1.34 - 1.42 (m, 3H), 1.39 (s, 9H), 1.45 - 1.53 (m, 2H), 1.60 - 1.86 (m, 3H), 2.80 - 2.90 (m, 1 H), 3.08 - 3.53 (m, 4H). LCMS (**Method D**): m/z 215 (M+H)^+^ (ES+), at 1.53 min, UV inactive. [a]D^20^ = + 21.3 (c = 1.0 in MeOH)

**STEP 3**. Methyl cyclopent-3-ene-1-carboxylate (4.42 g, 35 mmol) was dissolved in DCM/MeOH (160 ml, 3: 1) and cooled to −78°C. Ozone was passed through the solution until a blue color persisted. Excess ozone was purged from the reaction mixture with dry N_2_. Dimethylsulfide (10 ml) was added and the reaction mixture was warmed to rt, the solvent was removed *in vacuo*. The residue was added to a solution of *tert*-butyl (4S)-4-aminoazepane-1-carboxylate (7.5 g, 35 mmol), STAB (18.57 g, 87.6 mmol), NEt_3_ (4.26 g, 42.1 mmol) and acetic acid (1.8 ml_) in DCE (200 ml). The mixture was stirred for 3 hours at rt and was then poured into aqueous Na_2_C0_3_ solution. The mixture was extracted with EtOAc (3 × 200 ml), the organic phase was washed with water (100 ml) and brine (100 ml), dried over Na_2_S0_4_. The solvent was removed *in vacuo*, and the residue was purified by column chromatography (gradient 0% to 25% EtOAc in Petroleum ether) to give *tert*-butyl (4S)-4-[4-(methoxycarbonyl)piperidin-1-yl]azepane-1-carboxylate (7.7 g, 64.6% yield) as a yellow oil. LCMS (**Method C**): m/z 341 (M+H)^+^ (ES+), at 1.49 min, UV inactive.

**STEP 4**. *tert*-Butyl (4S)-4-[4-(methoxycarbonyl)piperidin-1-yl]azepane-1-carboxylate (7.7 g, 22.7 mmol) was dissolved in THF and water (60 ml, 1: 1) and cooled to 0°C. NaOH (1.0 g, 24.5 mmol) was added and the reaction mixture was stirred at rt for 3 h. The organics solvents were removed *in vacuo*, and the aqueous phase was acidified with acetic acid to pH = 3~4, then concentrated to dryness. The residue was suspended in CHCI3 (40 ml) and filtered to remove inorganic salts. The filtrate was evaporated to dryness to afford 1-{(4S)-1-[(*tert*-butoxycarbonyl)carbonyl]azepan-4-yl}piperidine-4-carboxylic acid (6.2 g, 84% yield) as a yellow oil. 1H NMR: (400MHz, DMSO-d6) δ: 1.38 (s, 9H), 1.45 - 1.53 (m, 6H), 1.70 - 1.78 (m, 4H), 2.08 - 2.20 (m, 3H), 2.35 - 2.42 (m, 1 H), 2.65 - 2.69 (m, 2H), 3.12 - 3.19 (m, 2H), 3.30 - 3.41 (m, 2H), 8.32 (br. s, 1 H) LCMS (**Method C**): m/z 327 (M+H)^+^ (ES+), at 1.35 min, UV inactive [a]D^20^ = + 1 1.0 (c = 1.8 in MeOH)

**STEP 5**. 1-{(4S)-1-[(*tert*-butoxycarbonyl)carbonyl]azepan-4-yl}piperidine-4-carboxylic acid (2.0 g, 6.135 mmol) was dissolved in DMF (25 mL) and cooled to 0°C. (1-methylcyclobutyl)amine. HCl (1.49 g, 0.18 mL, 12.27 mmol), HATU (3.5 g, 9.202 mmol) and DIPEA (3.67 g, 5.34 mL, 30.67 mmol) were added. The reaction mixture was stirred at rt for 16 h and the solvents were removed *in vacuo*. The residue was partitioned between DCM and sat. NaHCO_3_ sol., the organic layer was washed with sat. NaCI sol. and dried (MgSO_4_). The solvents were removed *in vacuo*, and the residue was purified by column chromatography (normal phase, [Biotage SNAP cartridge KP-sil 50 g, gradient 0% to 10% MeOH in DCM]) to give *tert*-butyl (S)-4-(4-((1-methylcyclobutyl)carbamoyl)piperidin-1-yl)azepane-1-carboxylate (0.459 g, 19%) as a pale yellow gum. LCMS (**Method A**): m/z 394 (M+H)^+^ (ES^+^), at 1.81 min, UV inactive

**STEP 6**. *tert*-Butyl (S)-4-(4-((1-methylcyclobutyl)carbamoyl)piperidin-1-yl)azepane-1-carboxylate (459mg, 1.14 mmol) was dissolved in DCM (4 mL) and TFA (2 mL) was added. The reaction mixture was stirred at rt for 4h under nitrogen, then the solvents were removed *in vacuo*. The residue was dissolved in DCM (10 mL) at rt. NEt_3_ (0.49 mL, 3.50 mmol) and ethyl chloroformate (0.22 mL, 2.33 mmol) were added and the reaction mixture was stirred at rt overnight under nitrogen. The solvents were removed *in vacuo*, and the residue was partitioned between DCM and sat. NaHCO_3_ sol.), organic layer washed with sat. NaCI sol. and dried over MgSO_4_. The residue was purified by column chromatography (normal phase, [Biotage SNAP cartridge KP-sil 25 g, gradient 3% to 10% MeOH in DCM]) to give ethyl (S)-4-(4-((1-methylcyclobutyl)carbamoyl)piperidin-1-yl)azepane-1-carboxylate (166 mg, 39%) as a yellow gum. 1H NMR: (400MHz, DMSO-d6) d: 1.13 (td, J = 7.1, 2.3, 3H), 1.28 (s, 3H), 1.29 - 1.60 (m,7H), 1.60 - 1.87 (m, 7H), 1.90 − 1.99 (m, 1H), 2.02 − 2.24 (m, 4H), 2.32-2.40 (m, 1H), 2.65 − 2.73 (m, 2H), 3.06 − 3.22 (m, 2H), 3.35 − 3.48 (m, 2H), 3.98 (qd, J = 7.0, 2.7, 2H), 7.68 (br.s, 1H). LCMS (**Method B**): m/z 366 (M+H)^+^ (ES^+^), at 3.28 min, UV inactive.

### Summary of synthesis of HTL9936

**Figure:**
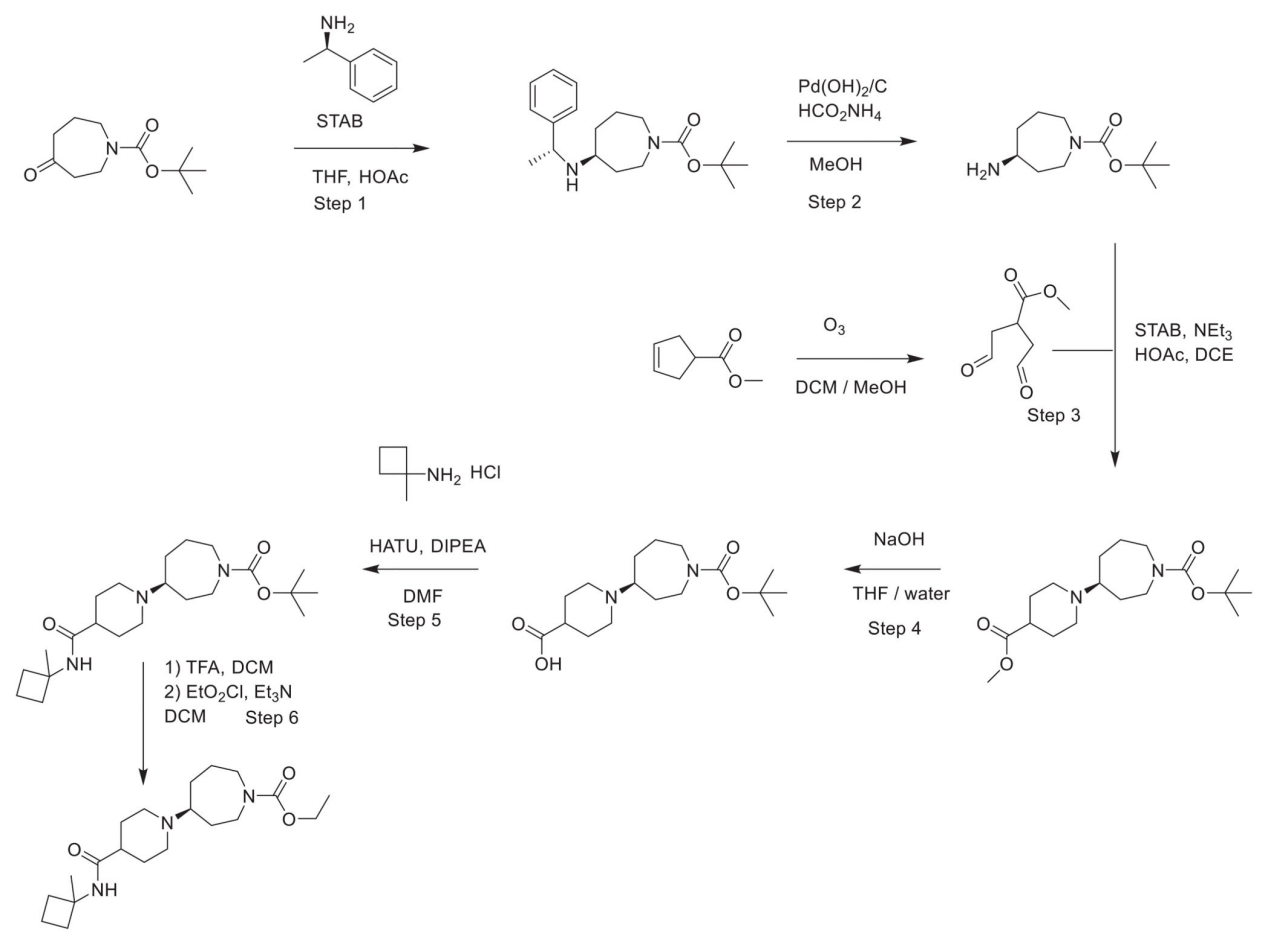


### IP1 accumulation assay performed on CHO-FlpIn cells expressing the M1-receptor

CHO-FlpIn cells stably expressing human M1-WT were grown to confluence in T75cm^2^ flasks in Ham’s F-12 media containing 10% fetal bovine serum and 1% penicillin/streptomycin and under hygromycin B selection (400 μg/mL). Cells were harvested and centrifuged at 1000 *x*g for 3 min prior to resuspension in 1X stimulation buffer ((in mM): HEPEs, 10; CaCl_2_, 1; MgCl_2_, 0.5; KCl, 4.2; NaCl, 146; glucose, 5.5; LiCl, 50; pH7.4) at 1.43 ×10^6^ cells/mL. Test compounds (7 μl/well) and cell suspension (7 μl/well) were added to 384-well white proxiplates (PerkinElmer). Following a brief centrifugation, plates were incubated at 37°C for 45 min. The IP1-d2 conjugate and the anti-IP1 cryptate Tb conjugate (IP-One Tb assay kit, CisBio) were diluted 1:30 in lysis buffer and 3 μL of each were added to each well. The plate was incubated at 37° C for 1h and FRET between d2-conjugated IP1 (emission at 665 nm) and Lumi4-Tb cryptate conjugated anti-IP1 antibody (emission at 620 nm) was detected using an Envision plate reader (PerkinElmer). Results were calculated from the 665/620 nm ratio and normalized to the maximum response stimulated by ACh.

### IP1 accumulation assay performed on HEK293T cells expressing the M1-receptor

HEK293T cells transiently transfected with a plasmid encoding the human M1 receptor were plated in poly-D-lysine coated 96-well plates at cell density of 40,000 cells/well. After 24h in culture, the plates were washed and incubated in Hank’s Balanced Salt Solution supplemented with 20mM HEPES (HBSS) for 30 min. The HBSS was then replaced with assay buffer containing HBSS supplemented with 30mM LiCl and test compounds were added. Plates were then incubated at 37°C for one hour. The assay buffer was removed, and cells were lysed through the addition of 40 μl/well of IP-One Assay Kit lysis buffer (CisBio) followed by a 10 min shaking incubation at room temperature. The resulting lysates were assayed for IP-One using an HTRF IP-1 assay kit (CisBio) according to the manufacturer’s instructions. TRF emissions (665 nm and 620 nm) were measured using a Pherastar FS microplate reader (BMG Labtech).

### [^3^H]-NMS binding to the M1-receptor

#### Competition binding

Membranes (5 μg/tube) prepared from CHO-FlpIn cells expressing the M1-receptor were incubated with an approximate K_D_ concentration of [^3^H]-NMS (0.3 nM) and increasing concentrations of HTL9936 (0.1 nM to 300 μM) for 1 hour at 37°C in binding buffer containing 100 mM NaCl, 10 mM MgCl_2_, and 20 mM HEPES, pH 7.4. Non-specific binding (NSB) was determined in the presence of 1 μM atropine). Reactions were terminated by rapid filtration onto GF/B filter paper (Whatman, Maidstone, UK) and three washes with 3 mL ice-cold 0.9% NaCl using a Brandel harvester (M-24TI; Brandel, Fort Lauderdale, FL). Membrane bound radioactivity was determined by liquid scintillation (Ultima Gold; PerkinElmer) counting.

#### Kinetic binding

For determination of [^3^H]-NMS dissociation kinetics, membranes (5μg/tube) expressing the M1-receptor were pre-incubated with [^3^H]-NMS for 1 hour at 37°C in binding buffer containing 100 mM NaCl, 10 mM MgCl_2_, and 20 nM HEPES, pH 7.4. Dissociation of the bound radioligand was initiated by the addition of atropine (10 μM) alone or atropine (10 μM) plus 100 μM HTL-9936 added in a reverse time course protocol. Reactions were terminated by rapid filtration onto GF/B filter paper (Whatman, Maidstone, UK) and three washes with 3 mL ice-cold 0.9% NaCl using a Brandel harvester (M-24TI; Brandel, Fort Lauderdale, FL). Membrane bound radioactivity was determined by liquid scintillation (UltimaGold; PerkinElmer) counting.

### pERK1/2 assay on CHO-K1 cells expressing muscarinic receptors

Functional assays were performed using the Alphascreen Surefire phopho-ERK1/2 assay. CHO-K1 cells stably expressing the human muscarinic M1, M2, M3, or M4 receptor or M1/M4 W101A γ12.1 StaR were plated (25K/well) onto 96-well tissue culture plates in MEM-alpha + 10% dialysed FBS. Once adhered, cells were serum starved overnight. Agonist stimulation was performed by the addition of 5 ml agonist to the cells for a pre-optimized 5 min period (37°C). Media was removed and 50 μl of lysis buffer added. After 15 min a 4 μl sample was transferred to a 384-well plate and 7 μl of detection mixture added. Plates were incubated for 2 h with gentle agitation in the dark and then read on a PHERAstar plate reader. pEC_50_ and %E_max_ values were estimated from the resulting data for each receptor subtype using GraphPad Prism.

### pERK assay on CHO-K1 cells expressing muscarinic receptors

HEK293T cells transiently transfected with a plasmid encoding the human M1 receptor were plated in poly-D lysine coated 96-well plates at a density of 40,000 cells/well. After 48 h in culture, stimulation of ERK protein phosphorylation at Thr202/Tyr204 was assessed using a homogeneous time resolved FRET (HTRF) Phospho-ERK (Thr202/Tyr204) detection kit (Cisbio). Confluent mono-layers of the cells were serum-starved for 7 hours before the experiment. The cells were washed in 200 μL PBS and then incubated in serum-free DMEM medium at 37°C. Cells were stimulated with acetylcholine or HTL 936 for 5 min at 37°C. The stimulations were terminated by aspiration of the test compounds and the addition of 50 μL lysis buffer supplemented with blocking reagent. Lysates were gently agitated (600 rpm) at room temperature for 10 min. Subsequently, 16 μL of lysate was transferred to a 384-well white ProxiPlate (PerkinElmer) and incubated with 4 μL premixed antibody solution (anti-phospho-ERK1/2 Cryptate and anti-phospho-ERK1/2) for 2 hours at room temperature with agitation at 700 rpm. Time resolved fluorescence emission (665 nm and 620 nm) was determined using a CLARIOstar microplate reader (BMG Labtech).

### Arrestin interaction assay with the M1-receptor

To assess arrestin recruitment to M1-receptor a bystander BRET assay was employed (Namkung et al., 2016). HEK293T cells were co-transfected with plasmids encoding: 1. human β-Arrestin-2 fused at its N terminus to Nanoluciferase (Nluc); 2. the mNeonGreen (mNG) fluorescent protein fused at its C terminus with the prenylation CAAX sequence of KRas; and 3. a plasmid encoding human M1. For these transfections a DNA weight ratio of 1:25:5 was used for the Nluc-β-Arrestin-2: mNG-CAAX: M1-WT plasmids. After transfection, cells were cultured 24h before 20,000 cells/well were transferred to white poly-D-lysine coated 96-well plates and cultured a further 24h. For the assay, the white 96-well plates were washed twice with HBSS and incubated for 30 min at 37°C. Nluc substrate, Coelenterazine 400a, was then added to a final concentration of 5 μM and incubated for 15 min. Dual 535 and 475 nm luminescent emission measurements were then taken at 1.5 min intervals using a PherStar FS plate reader (BMG labtech) for 6 min prior to and 30 min following the addition of the indicated test compounds. Net BRET responses were calculated as the 535/475 ratio after correcting for both the well baseline and test compound vehicle (0.1% DMSO) response. BRET data were then reported as the area under the Net BRET curve for the 30 min following test compound addition, with responses for each experiment normalized to the maximal response to Ach in that experiment.

### Determination of the internalisation of the M1-receptor

Internalization of the M1-receptor was assessed using a bystander BRET assay designed to measure the translocation of M1 receptor to early endosomes (Namkung et al., 2016). HEK293T cells were co-transfected with: 1. a plasmid encoding human M1 fused at its C-terminal to Nluc; and 2. a plasmid encoding mNG fused at its C-terminal to the FYVE domain of endofin. A DNA weight transfection ratio of 1:2 was used for Nluc tagged M1 receptor to mNG-FYVE. 24 h after transfection, 50,000 cells/well were transferred to white 96-well plates and cultured a further 24 h prior to the assay. Plates were then washed with HBSS, before incubating in HBSS for 30 min at 37°C. Coelenterazine 400a was added to a final concentration of 5 μM and plates incubated a further 15 min. BRET measurements were taken using a PherStar FS plate reader, measuring luminescent emission at 535 and 475 nm at 2 min intervals for 6 min prior to addition of test compounds, and a further 1 h after addition of test compounds. Net BRET responses were calculated as the 535/475 ratio after correcting for both the well baseline and test compound vehicle response. BRET data were then measured as the area under the Net BRET curve for the 60 min following test compound addition, which was then normalized to the maximal response to Ach in each assay.

### Activation of G-proteins G_q_/G_11_/G_15_

Direct activation of members of the Gq/11 family by M1-receptor was assessed using the ‘TruPath’ BRET G protein biosensors (Olsen et al., 2020)Cells were transfected with a 1:1:1:1 ratio of plasmids encoding: 1. human M1, 2. Gq, G11, or G15 with an internal Rluc2 tag as described in (Olsen et al., 2020). 3. G γ 9 (for Gq studies) or Gγ13 (for G11 and G15 studies), and 4. Gβ3. 24 h after transfection cells were transferred to poly-D-lysine coated white 96 well plates and cultured a further 24h prior to the assay. Cells were then washed and incubated in HBSS for 30 min. Coelenterazine 400a was added to a final concentration of 5 μM and test compounds were added to the plate. Four minutes later bioluminescent emissions at 485-90nm and 525-90nm were measured using a CLAR-IOstar microplate reader (BMG Labtech). The BRET ratio of 525/485 emission was taken and responses were expressed as a percentages of the maximal BRET response obtained to Ach.

### GTPγS assay to establish G protein activation by the M1-receptor

Wild-type or M1-KO mice (Bradley et al., 2017) (8-12 weeks) were humanely killed and cortical tissue was dissected on ice. Tissue was suspended in ice-cold buffer A (containing 0.9% (w/v) NaCl, 10 mM HEPES, 0.2% (w/v) EDTA, pH 7.4) and homogenized (4 ×5s bursts) using a Polytron homogenizer. The suspension was centrifuged at 200 xg for 5 min at 4°C using an Eppendorf 5810R bench-top centrifuge. Supernatants were collected and re-homogenized as above. The suspension was subsequently centrifuged for 20 min at 40,000 xg at 4°C using a Beckman Coulter Avanti JXN-26 centrifuge with a JA-25.25 rotor. Supernatant was discarded, and the pellet was re-suspended in 10 mL ice-cold buffer B (10 mM HEPES, 10 mM EDTA, pH 7.4). The pellet was homogenized, GTP (1 mM final) was added and the suspension was incubated at 37° C for 15 min. The suspension was subsequently centrifuged for 20 min at 40,000 xg at 4°C and the pellet was re-suspended in 15 mL ice-cold buffer C (10 mM HEPES, 0.1 mM EDTA, pH 7.4) and re-homogenized as before. Suspension was centrifuged again for 20 min at 40,000 xg at 4°C. The final pellet was re-suspended in buffer C and protein concentration was estimated using a Bradford assay and this resuspended homogenate further diluted in buffer C to produce a concentration of 2 mg/mL. This preparation is the membrane preparation used in the [^35^S]-GTPγS binding assay described below.

[^35^S]-GTPγS binding and immunoprecipitation of Gα subunits was performed as previously described (Bradley et al., 2017). Specifically, wild-type or M1-KO membranes were diluted in assay buffer (in mM: HEPES, 10; NaCI, 100; MgCl2, 10; pH 7.4) containing a final concentration of 1 μM GDP. Membranes (75 μg in a total assay volume of 200 mL) were added to [^35^S]-GTPγS (1 μM final concentration) and agonists (CCh or CNO) and incubated at 30°C for 5 min. Reactions were terminated by the addition of 1 mL ice-cold assay buffer and immediate transfer to an ice bath. Samples were centrifuged (20,000 xg, 6 min, 4°C) and membrane pellets solubilised by the addition of 50 μL ice-cold solubilisation buffer (100 mM Tris HCI, 200 mM NaCI, 1 mM EDTA, 1.25% Igepal and 0.2% SDS, pH 7.4) and incubation for 1 h at 4°C on a shaking platform. Following complete protein re- solubilisation, 50 mL of solubilisation buffer without SDS was added. Solubilised protein was pre-cleared using normal rabbit serum at a dilution of 1:100 and 3% (w/v) protein A-Sepharose beads in TE buffer (10 mM Tris HCI, 10 mM EDTA, pH 8.0) added for 60 min at 4°C. Protein A- Sepharose beads and insoluble material were collected by centrifugation (20,000 xg, 6 min, 4°C) and 100 μL of the supernatant was transferred to fresh tubes containing Gq-specific anti-serum (Santa Cruz; sc393) and incubated overnight at 4°C. Protein A-Sepharose beads were added to samples, vortex mixed and rotated at 4°C for 90 min before being centrifuged (10,000 xg, 1 min, 4°C). Supernatants were aspirated and the protein A- Sepharose beads washed three times with ice-cold solubilisation buffer (without SDS). Recovered beads were then mixed with 1 mL FloScint-IV scintillation cocktail and counted by liquid scintillation spectrometry.

### Pharmacokinetic analyses of HTL9936 in animals undergoing fear conditioning

Compounds were administered via intraperitoneal injection (in 5% glucose) 30 min prior to blood collection. Mice were anesthetized with 3% isoflurane (2 L/min), and blood was collected by cardiac puncture of the left ventricle. Blood was immediately transferred to EDTA tubes and centrifuged at 1000 g for 10 min at 4°C; the supernatant was collected and frozen. Brains from each mouse were also dissected and snap-frozen on dry ice.

Brain samples were homogenized in three volumes of methanol/water [1:4 (v/v)] by weight. A 25 mL aliquot of each study sample, calibration standard, and control sample was added to a 96-well plate and mixed with 180 mL acetonitrile/methanol [1:1 (v/v)] containing internal standard. The samples were subsequently centrifuged, and the resulting supernatants were diluted 12.5-fold with methanol/water [1:1 (v/v)] prior to analyzing 10μl aliquots by liquid chromatography−tandem mass spectrometry as previously described (Bradley et al., 2017).

### Novel object recognition behavioral test

Male adult Wistar rats (Harlan, UK; approximately 11 weeks of age) were handled for two days prior to the experiment. Rats were administered vehicle (acidified saline) or HTL9936 (3, 10 or 30 mg/kg) by oral gavage 90 min prior to training. Donepezil (0.1 mg/kg) and galanthamine (3 mg/kg) were administered via intraperitoneal injection (in saline) 60 min prior to training. Following a 10-min habituation period, each rat was placed into the test arena comprising an open-field arena in the presence of two identical plastic shapes and allowed to explore for 5 min. The rat was then returned to its home cage. After 24 hours, the rat was returned to the test arena in the presence of one familiar object, and one novel object. The time exploring each object was recorded using image analysis software (Ethovision XT, Noldus, UK).

### Passive avoidance test behavioral test

Male adult Wistar rats (Harlan, UK; approximately 11 weeks of age) were acclimatised to the experimental rooms for three days (study days 1-3) and handled for a further two days (study days 4 and 5) prior to the experiment.

On study day 6, rats were administered vehicle (acidified saline) or HTL9936 (3, 10 or 30 mg/kg) by oral gavage 90 min prior to training. Donepezil (0.01 and 0.1 mg/kg) was administered via intraperitoneal injection (in saline) 60 min prior to training. Rats were trained in a single-trial, step-through, light-dark passive avoidance paradigm. The training apparatus consisted of a chamber 300 mm in length, 260 mm wide, and 270 mm in height. The front and top were transparent, allowing the experimenter to observe the behavior of the animal inside the apparatus. The chamber was divided into two compartments, separated by a central shutter that contained a small opening (50 mm wide and 75 mm high) set close to the front of the chamber. The smaller of the compartments measured 90 mm in width and contained a low-light (~200 lux) illumination source. The larger compartment measured 210 mm in width and was not illuminated. The floor of this dark compartment consisted of a grid of 16 horizontal stainless-steel bars that were 5 mm in diameter and spaced 12.5 mm apart. A current generator supplied 0.75 mA to the grid floor, which was scrambled once every 0.5 s across the 16 bars.

Animals were placed facing the rear of the light compartment of the apparatus, immediately after which, spontaneous behavior was assessed. The timer was started once the animal completely turned to face the front of the chamber. Latency to enter the dark chamber was recorded, and having completely entered the dark compartment foot shock was administered to the animal, which immediately returned to the light compartment. Animals were then returned to their home cages. Between each training session, both compartments of the chamber were cleaned to remove any confounding olfactory cues. Vehicle (saline) or scopolamine (1 mg/kg) was administered to rats 6 hours after training. Recall of the inhibitory stimulus was evaluated 24 hour post-training on study day 7, by returning the animal into the light chamber and recording their latency to enter the dark chamber, a criterion time of 600 s was employed. Observation of the animals was made by overhead video camera linked to a monitor behind a screened area. Animal treatment was blinded to the observer.

### Open field behavior behavioral test

During the rat passive avoidance testing, open field exploratory behavior was measured on study days 4 and 5 (acclimatisation and handling), 6 (training) and 7 (recall). The open field apparatus consisted of black-painted Perspex 620 mm^2^ with walls 300 mm high. Locomotor activity, measured as the distance traveled in a 300 s period, as well as incidences of rearing and grooming were recorded, using Ethovision XT tracking software (Noldus UK).

### Fear conditioning behavioral test

C57BL/6J male mice (aged 8−12 weeks) were acclimatized to the behavioral testing suite at least 2 hours prior to the test. Mice were injected (intraperitoneally) with vehicle (5% glucose) or scopolamine (1.5 mg/kg, (i.p.)) alone or in combination with HTL9936 or donepezil at the doses indicated (i.p.) HTL9936 30 min prior to training. Mice were placed in the conditioning chamber (ANY-maze Fear Conditioning System; Stoelting, Dublin, Ireland); after a 2-min adaptation period, they received three tone/foot shock pairings where the foot shock (un-conditioned stimulus; 2 s; 0.4 mA) always co-terminated with a tone (conditioned stimulus; 2.8 kH; 85 dB; 30 s). The conditioned stimulus−unconditioned stimulus pairings were separated by 1-min intervals. After the mice completed training, they remained in the conditioning chamber for 1 min and were then returned to their home cages. The next day, the mice were placed back in the conditioning chamber, and time spent immobile was recorded for 3 min to assess context-dependent learning. Data were analyzed using ANY-maze software (Stoelting).

### Prion-infection of mice

Tg37 hemizygous mice were inoculated by intracerebral injection into the right parietal lobe with 1% brain homogenate of RML prions aged 3 to 4 weeks as described previously (Bradley et al., 2017). Control mice received 1% normal brain homogenate (NBH).

### Delayed non-matching to position task (DNMP)

The effects of HTL9936 on performance in a delayed non-matching to position (DNMP) task was conducted in aged Beagle dogs (aged > 8 years of age). Animals were acclimatised in the testing facility for at least 3 months. During baseline days (days −12 to −8), 50 dogs underwent DNMP testing once daily using delays of 20 and 90 s equally dispersed over 12 trials per session. Mean performance over these 5 sessions was used to select 40 subjects that demonstrated both consistent responses and low levels of performance accuracy for subsequent testing.

These 40 subjects were tested on the DNMP using delays of 5, 55 and 105 s equally dispersed over 18 trials per session over five once-daily sessions (days −6 to −2). Therefore, baseline testing was conducted for a total of 10 days.

These dogs were then allocated into 5 groups, balanced for mean baseline performance accuracy at the longest delays (n = 8 per group). Treatment groups were vehicle (20% PEG400 in saline; s.c.), HTL9936 (0.3, 1 and 2 mg/kg; s.c.) or donepezil (1.5 mg/kg; p.o.; saline). Daily treatment administration began on day 0, and dogs were tested once daily on the DNMP at the same delays employed at baseline for a minimum of 10 days. Each test consisted of two phases; in phase one, the dog is presented with an object over one of the three food wells. After the dog moves the object and obtains the food reward beneath, the tray is removed from the dog’s sight and the delay is initiated. The second phase occurs after the delay and consists of presenting the dog with two identical objects to that used in the first phase. One is in the same position as the first phase and the other covers a food reward in one of the two remaining food wells.

### Quantitative EEG (qEEG) resting state in macaques

6 adult male Cynomolgus macaques (Macaca fasicularis; 8-10 kg; 12-15 years old) were used for EEG studies. These animals had been used previously on other studies and were not considered to be pharmacologically naive. Subjects were housed in a temperature-controlled recording room and are maintained on a 12-hour light/dark cycle. The animals had access to water *ad libitum* and were fed a full daily regimen of food each day (Purina Animal Nutrition, Gray Summit, MO) enriched with fresh fruit and/or vegetables. All studies are conducted in compliance with USDA, NIH and SRI International IACUC guidelines. SRI International is an AAALAC accredited institution. Animals were instrumented with an intra-abdominal telemetric EEG device (D70-EEE; DSI, St. Paul, MN) containing three biopotentials implanted in fully anesthetized animals by trained surgeons using a stereotactic frame over the left frontal cortex (Fp2; AP:+18.0mm ML:-15.0mm), central midline (Cz; AP:0.0mm ML:0.0mm) and posterior (P3; AP:-18.0mm ML:-16.0mm) locations and referenced to the occipital midline (Oz).. On the day of dosing, animals were acclimatised in the recording chambers for approximately 5-10 min. Following the acclimation period, animals were dosed subcutaneously with vehicle or compound and EEG activity was recorded for 120 min. All EEG recordings were acquired at 2 kHz in DataQuest ART (Version 4.3) software (DSI, St Paul, MN).

### Rat cardiovascular study of the effects of HTL9936 administration

HTL9936 or vehicle were administered by gavage (4mL/kg) at time = 0. Summary profiles were generated representing the mean of HTL9936 across 4-6 animals at 3, 10, 30 and 100 mg/kg on heart rate and mean arterial blood pressure. Arterial blood pressure and heart rate (determined from the pulsed blood pressure signal) were continuously measured 60 min before administration of HTL9936 or vehicle. At each time point the results were averaged over the preceding 5 min period. We performed pharmacokinetic analysis on a subset of the same animals used for the cardiovascular assessment on completion of all cardiovascular assessments (Table SIb).

### Dog intravenous infusion GLP cardiovascular study

Animals were implanted with calibrated sensors (DSI D70 Series) for arterial blood pressure (ABP). The signals were processed by the Open Art/ PoNeMah data acquisition/analysis software. Vehicle and test article were administered by intravenous infusion over 2 hours, using a constant dose volume of 10 mL/kg (5 mL/kg/hr) and data was continuously recorded at logging rates of 1 min. Two pre-dose readings of the heart rate, blood pressure (Diastolic, systolic and mean arterial pressure) and ECG intervals (PR-, QRS-, QT- and QTCF) were taken 15 min apart before administration of each dose. Following the start of the infusion, of vehicle or test article, readings were taken from the line averages at 15, 30, 45, 60, 75, 90, 105 and 120 min (i.e during the infusion), 150 and 180 min (i.e., 0.5 and 1 hour post infusion in sling) (additional recordings were continued to 12hours post-dose). The plasma concentrations of HTL9936 was determined in these experiments (Table Sc). Time given as minutes after the end of a 2hr intravenous infusion where 0 min indicates the end of the infusion period. Blood samples of approximately 2 mL were taken from the jugular vein of each animal at the end of infusion and again at the end of the 1 hour post-infusion observation period in sling following administration of vehicle and HTL9936.

### Clinical drug administration

For the healthy human volunteer single and multiple ascending studies HTL9936 oral solution was administered in a 100mL dose volume in amber, Type III glass bottles..HTL9936 was dissolved (low dose) or dispersed (high dose) in water and the solution adjusted to pH 5.2 ± 0.25. For intravenous (IV) administration a sterile 10mg/mL solution of HTL9936 was generate in citrate buffer (pH range5.5 ± 1). The required amount of sterile solution was subsequently injected into the infusion bag containing sodium chloride 0.9%w/v infusion fluid to provide the required dose for IV administration.

### Clinical EEG recordings

Routine EEG was performed for all subjects during screening enrolled in the multiple ascending dose study to avoid enrolling subjects showing clinically significant abnormalities. 19-lead EEG recordings were employed for quantitative (qEEG) analyses. qEEGs were are recorded in resting state with alternating 30 s eyes open and 30 s eyes closed for 5 min first and then about 5 min eyes closed. The P50 protocol employed a series of “click pairs.” The interval between click pairs is at random between 6 to 10 s (Stimuli S1 and S2, 1000 Hz, duration 3 ms, 80 dB sound pressure level [SPL], stimulus onset asynchrony [SOA] = 500 ms, The MMN protocol used twelve different frequencies ranging in 50 Hz steps from 700 to 1250 Hz delivered binaurally via special headphones or earphones. All stimuli were presented with a constant SOA of 300 ms in a pseudo-randomized order assuming a frequency deviation of one train to another of at least 100 Hz with three different lengths/durations of series which occurred evenly. For the P300 active auditory oddball paradigm sinusoidal tones of 90 dB SPL intensity and 50 ms duration were delivered binaurally in an inter-stimulus-interval of 1000 msec. The standard stimulus, a low frequency tone (1000 Hz) appeared with a frequency of 85%. The target stimulus (higher frequency tone of 2000 Hz) with a frequency of 15% was presented in a pseudo-randomized manner and in a way that it was never repeated immediately. The participant was instructed to press a corresponding button as quickly as they can after they recognized the target stimulus (high tone).

### Clinical functional MRI

All functional MRI images were acquired on a 3T scanner using gradient echo-echo planar imaging (GRE-EPI: TR: 300ms; TE: 30 ms; 90-degree flip angle; slices: 53; voxel size 3.75x 3.75 mm) and an 8-channel head coil. All data processing was done using Statistical Parameteric Mapping (SPM) version 5 software. All functional images were co-registered to subjects own T1 weighted structural image for anatomical localization, realigned using a least-squares approach and a 6 parameter (rigid body) spatial translation, normalized to Montreal neurological institute (MNI) using a high dimensional, non-linear transformation and smoothed using 3D 7 mm Gaussian filter. A high pass filter was applied to remove low temporal frequency drift. Bilateral hippocampal and caudate masks based on previous publications using the Arena task were used for analysis (Antonova et al., 2009, 2011). The masks consisted of spheres of radius 6mm centered on co-ordinates from these papers.

## Quantification and Statistical Analyses

### Radioligand Binding

Data was analyzed using PRISM (GraphPad). For saturation binding, non-specific and total binding data were analyzed to a one site binding model. DPM was converted to fmol/mg to compare across constructs. Inhibition binding curves between [^3^H] 77-LH-28-1 and test compound were fitted to a one-site binding model. The Cheng Prusoff equation was used to estimate pK_i_ (Cheng and Prusoff, 1973).

### Crystallography Data processing

X-ray diffraction data were measured on a Pilatus 6M detector at beamline I24 (Diamond Light Source). Complete datasets for M1_StaR_-HTL9936 or for M1StaR-GSK1034702 were acquired using diffraction data from six different crystals each whereas data from four crystals were assembled for M1-StaR-77-LH-28-1. Data from individual crystals were integrated using *XDS* (Kabsch, 2010) or *MOSFLM* (Battye et al., 2011) combined using *POINTLESS* (EVANS, 2006) from the *CCP4 suite* (Winn et al., 2011) and merged and scaled using *AIMLESS* (Evans and Murshudov, 2013) and the *STARANISO* procedure. Data collection statistics are reported in Table S2.

### Structure solution and refinement

The structures of the M1-StaR-T4L-ligand complexes were solved by molecular replacement (MR) with *Phaser* (McCoy et al., 2007) using the structure of Turkey Beta1 adrenergic receptor in complex with Dobutamine (Warne et al., 2011) as the search model (PDB code: 2Y00). Iterative rounds of model refinement performed using *BUSTER*, were interspersed with manual model building in *COOT (Emsley et al*., 2010). 2 TLS groups corresponding to the receptor and to the T4-lysozyme respectively were defined during refinement. The final models were validated using *MolProbity* (Chen et al., 2010). The final refinement statistics are presented in Table S2. Structure figures were generated using *PyMOL*.

### Molecular dynamics simulations

Molecular dynamics (MD) simulations were run for M_1_ in complex with tiotropium (PDB 5XCV) (Thal et al., 2016), HTL9936, GSK1034702 or 77-LH-28-1. For M_1_ in complex with tiotropium the cocrystallized FLAG peptide bound to the intracellular side of the receptor was included. Input PDB files were processed with Schrödinger Maestro’s Protein Preparation tool (2020-3) by adding hydrogen atoms, modeling missing side chains, and determining the most relevant protonation states of residues and ligands at pH 7.4. Fusion proteins inserted in the ICL3 of the constructs were removed and the long unstructured loop was truncated with a tetraglycine region between the Ballesteros-Weinstein positions 5.68-6.24, following similar approaches by Dror et al. (Dror et al., 2009) and Miszta et al. (Miszta et al., 2018). The constructs were embedded in POPC phospholipids and solvated in TIP3P water and 150 mM NaCl. The systems were parameterised using OPLS3e (Roos et al., 2019) after optimization of the parameters of the ligands using Maestro’s Force Field Builder tool. Desmond 2020-3 was used for the MD simulations. Relaxation and equilibration to 300 K and 1 bar were run using the standard protocol for membrane proteins. Production runs were performed for 100 ns at 300 K and 1 bar in the NPT ensemble. Temperature and pressure were controlled with Nose-Hoover chain thermostat (Hoover, 1985) and the semiisotropic MTK barostat. The RESPA integrator was employed with timesteps of 2 fs, 2 fs and 6 fs for bonded, short-range nonbonded and long-range nonbonded interactions, respectively.

### Extracellular cavity volume analysis

The volume of the extracellular cavity of the proteins throughout the MD simulations was computed using Epock (Laurent et al., 2015) using the same cuboid box after alignment of the frames to a common reference.

### Ligand Interaction Fingerprints

Interactions between ligands and receptors over the MD simulations were calculated using Schrodinger’s Python API. Detected contacts were classified as either “polar” or “hydrophobic” and averaged over the MD simulations to obtain estimates of the occupancy of the interactions.

### Bias factor calculations

Bias factors were calculated by fitting an operation model (van der Westhuizen et al., 2014) to all concentration response data in order to obtain τ/K_A_ values for ACh and HTL-9936 in each assay. Δτ/KA_HTL-9936_ values were obtained by subtracting the τ/K_A_ obtained for Ach from the value for HTL-9936 in the same assay. ΔΔ τ/K_A_ values were obtained by calculating the difference between the Δ τ/KA_HTL-9936_ in each assay.

### Rat Cardiovascular Study

At each time point the results were averaged over the preceding 5 min period. For presentation only the 15min time points are presented Figure SIa,b and Table SIa. Data are expressed as mean ± SEM analyzed by repeated-measurement ANOVA and pairwise comparisons of each compound to vehicle by post hoc Dunnett’s test. Significant values are relative to the vehicle treatment only. Differences were considered to be significant by p < 0.05 for 10 mg/kg $, 30 mg/kg * and 100 mg/kg + respectively. Averaged time points over 0-300 min time period presented as mean ± SEM analyzed by ANOVA and pairwise comparisons of each compound to vehicle by post hoc Dunnett’s test. Significant values are relative to the vehicle treatment only. a p < 0.05, b p < 0.01, c p < 0.001.

### Dog intravenous infusion GLP cardiovascular study

For each variable, the mean of the pre-dose values recorded for each animal on each dose were taken as the baseline for that dose and that animal. Each time point was analyzed using ANOVA, fitting DOSE and ANIMAL as fixed effects with the baseline value as a covariate. Pairwise comparisons of each dose with control (Dose 1) were made using Dunnett’s test (Figure Sc,d).

### Non-human primate qEEG measurments

Data was analyzed using existing, customized MATLAB scripts with the EEGLAB toolbox. EEG traces were filtered using a finite impulse response windowed-sync filter with a Blackman window with the high-pass cutoff at 1Hz, with a 0.5Hz half amplitude transition band and the low-pass cutoff at 200Hz with a 10Hz transition band. Time frequency analysis was done by applying fast Fourier transform (FFT) was conducted on 4 s periods filtered by a sliding Hanning window with a 92.75% overlap to calculate power with a 0.25Hz frequency resolution. For each frequency band, outliers were defined as the median ± eight times the median absolute deviation over the window of interest (i.e., 2, 5 or 10 min), and removed from the subsequent analysis. Frequency bands were defined as follows: Delta: 1-4Hz, Theta: 4-8Hz, Alpha: 8-12Hz, Sigma: 12-16Hz, Beta: 16-24Hz, Low Gamma: 24-50Hz, High Gamma: 50-100Hz. Mean absolute power, log transformed absolute power, and relative power was calculated based on each frequency band’s contribution to the total absolute power (i.e., % power). The mean normalized power was subsequently calculated by dividing each frequency band’s absolute power in the drug condition with the corresponding power in the control condition for each individual animal (i.e., use each animal as its own control) and then average across subjects. Data was analyzed using a one-way repeated-measures ANOVA to identify treatment effects within a frequency band for all parameters followed by Fisher’s post hoc tests when applicable to identify individual differences and significance defined as p £ 0.05.

### Clinical EEG measurements

All EEG datasets were imported Into the Brainvision Analyzer software. The data was limited to the following 19 channels: Fp1, Fp2, F7, F3, Fz, F4, F8, T3, C3, Cz, C4, T4, T5, P3, Pz, P4, T6, O1 and O2. In addition, the vertical and horizontal EOG Channels (VEOG /HEOG) were retained for the purpose of ocular correction. Data was filtered with a band pass of 0.1 − 30 Hz for the P300 paradigm. A slope of 12 dB/octave was applied. For the QEEG analysis a high pass filter with an edge frequency of 1 Hz And 48 dB/octave was applied. The Gratton And Coles ocular correction algorithm was applied (Gratton et al., 1983) that is available in the Analyzer Software VEOG and HEOG were used as reference channels and removed after ocular correction. The P300 data was segmented into epochs of 600 ms duration including a pre-stimulus baseline of 100 ms that was subtracted from each segment individually.

Statistical analysis was carried out in MATLAB. Because of the small sample sizes of the groups (N = 4 Placebo; N = 6 Cohort 1/low exposure; N = 6 Cohort 2/high exposure) only exploratory statistical analyses was carried out using paired t tests for within group comparisons and independent t tests for between group comparisons. Within groups, the baseline Day (Day 1) was compared against Day 4 and Day 9. This was done for pre- and post-dose measurements separately.

### Clinical functional MRI

A block design was used to assess functional activation during encoding, retrieval, and rest (control) conditions of the Arena task. Beta-estimates of functional response during each condition were created by modeling signal changes convolved with a double gamma hemodynamic response function with peak responses at 4 and 8 s following stimulus onset for each individual. In the first level analysis, difference in beta estimates for encoding versus rest, and retrieval versus rest were derived. These images were used in a higher-level GLM analysis investigating treatment-group differences (placebo, 10.1 mg HTL0016878, and 40.3 mg HTL0016878) in functional activation during encoding and retrieval using study site as covariate. Treatment- group differences in functional response was considered significant if Z-values were greater than 3.2 with a small volume corrected cluster probably of p < 0.05. Functional responses in the a-priori ROIs were extracted using hippocampal, ventral striatum, and caudate masks derived from subject own T1 weighted image. To test for drug effects in these pre-specified regions an ANOVA including fixed-effect terms representing treatment group, study site as a between subject factor, and baseline MMSE score as covariate was used.

Sample size was determined on the basis of previous studies which have either assessed the behavioral cognitive effects of M1 active compounds in humans (Nathan et al., 2013; Snyder et al., 2005) or the effects of cholinergic compounds on BOLD signal (Antonova et al., 2011). These studies reported large effect sizes suggesting that setting the significance level to 0.05 (two tailed), 18 subjects would provide > 90% power of identifying a similar effect.

Image data was converted from DICOM file format to NIfTI file format, an agreed standard format to facilitate transfer between different packages. Preprocessing was performed using the Statistical Parameteric Mapping (SPM) analyses package. Images were A representative realigned image was used to derive parameters for spatial normalization to a standard template (Montreal neurological institute (MNI)) using a higher dimensional, non-linear transformation. The realigned time-series and spatially normalized images were smoothed with an appropriate Gaussian kernel. High pass temporal filtering will be used. A slice timing correction procedure was used to account for variation in slice time

## Supplementary Material

Supplemental figures

## Data Availability

Materials described in this manuscript may be made available to qualified, academic, non-commercial researchers through a materials transfer agreement upon request the lead contact Andrew Tobin (andrew.tobin@glasgow.ac.uk).

## Highlights

- Agonist:receptor co-structures inform synthesis of a M1-receptor partial agonist
- M1-receptor agonist shows pro-cognitive activity in preclinical models
- M1-receptor agonist shows low cholinergic adverse effects in human studies
- M1-receptor agonist activates centers of learning and memory in elderly volunteers

## Data and code availability

- The M1-StaR complex structure coordinates and structure factors are available via the Protein Data Bank, accession code (PDB: 6ZFZ, PDB: 6ZG4 and PDB: 6ZG9). Human Datasets generated have been filed in EUDRACT (EUDRACT.ema.europa.eu; ref: 2013-002307-34 and 2015-002490-38) and the U.S. National Library of Medicine (Clinicaltrails.gov ref: NCT02291783 and NCT02546310) and are not publicly available in accordance with regulations for Phase 1 data.
- This paper does not report original code.
- Any additional information required to reanalyze the data reported in this paper is available from the lead contact upon request.
