# Supplementary material for: From structure to clinic: Design of a muscarinic M1 receptor agonist with the potential to treat Alzheimer’s disease
Source: Cell. Author manuscript; Available in PMC 2024 Jul 11. (PMC7616177; doi:10.1016/j.cell.2021.11.001)
Supplement: Supplemental figures [file EMS197030-Supplementary-Supplemental_figures.pdf]

CellPress

Supplemental figures

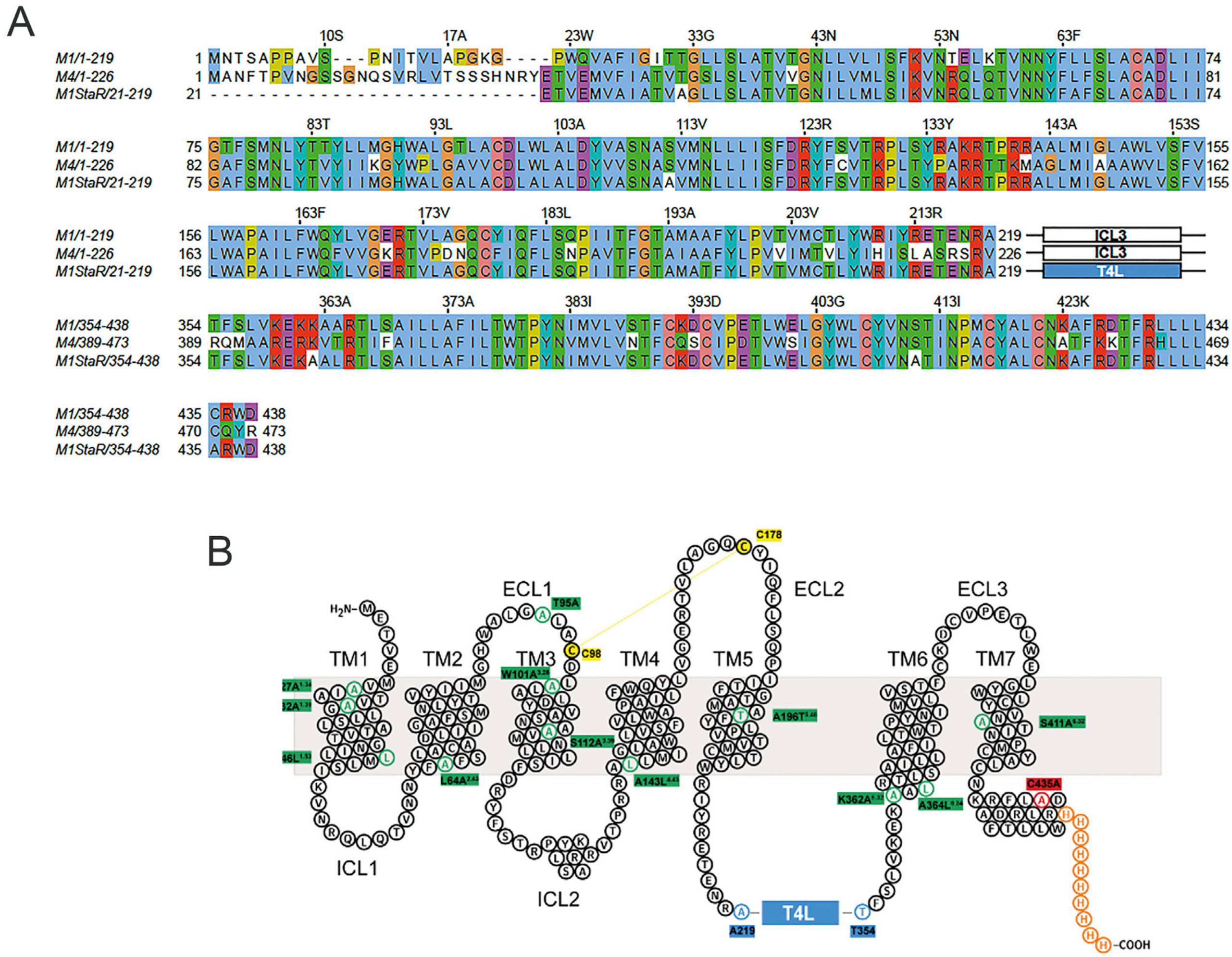

Figure S1. Crystallisation construct design and pharmacological characterization, related to Figures 2A–2C

(A) Sequence alignment across the human M1 and M4-receptors and the final M1-StaR. The ClustalX colouring scheme (as implemented in JALVIEW) was used in the alignment. (B) M1-StaR-T4L crystallisation construct in schematic snake-plot representation. Thermostabilising mutations (F27A1.34, T32A1.39, V46L1.53, L64A2.43, T95A, W101A3.28, S112A3.39, A143L4.43, A196T5.46, K362A6.32, A364L6.34, S411A7.46) are represented in green, whereas the C435A mutation to remove a post-translational modification is in red. The disulfide bond between C98 and Cys178 is shown as a yellow line.

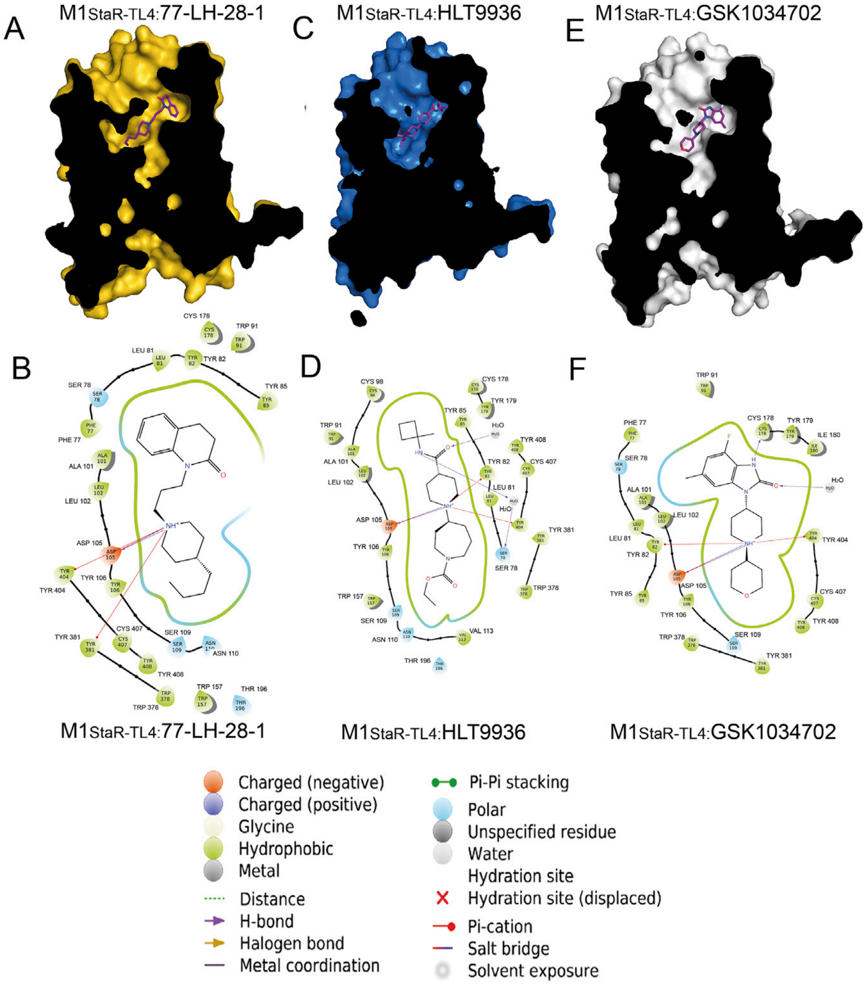

Figure S2. Molecular details of the M1-receptor extended orthosteric agonist binding site, related to Figures 2A–2C and 3A–3E

Surface representation of the M1-receptor as viewed parallel to the membrane plane to reveal the occupied binding sites and ligand interaction diagrams depicting the key molecular interactions made by (A,B) 77-LH-28-1, (B.C) HTL9936 and (C,D) GSK1034702.

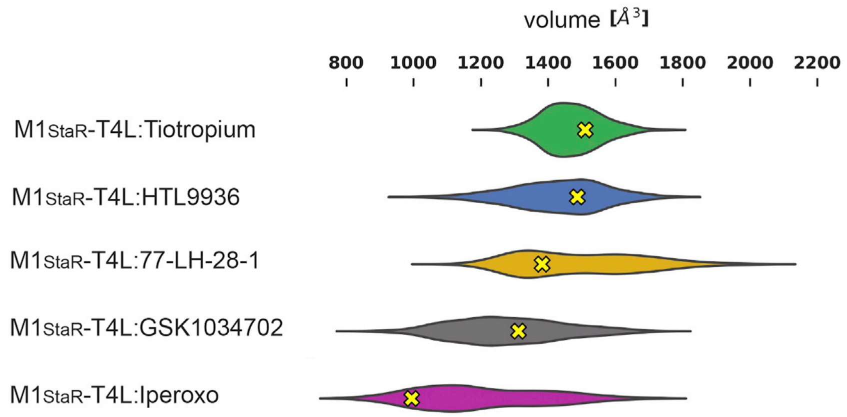

Figure S3. Molecular dynamic simulations of ligand binding to the M1-receptor, related to Figure 3F

Comparative analysis of ligand binding site volumes of crystal structures (yellow cross) and Molecular Dynamics (MD) simulations (violin plots) of M1-StaR-T4L bound to antagonist tiotropium (PDB:5XCV), partial agonist HTL9936, and agonists GSK1034702, 77-LH-28-1 and iperoxo (PDB:6OIJ).

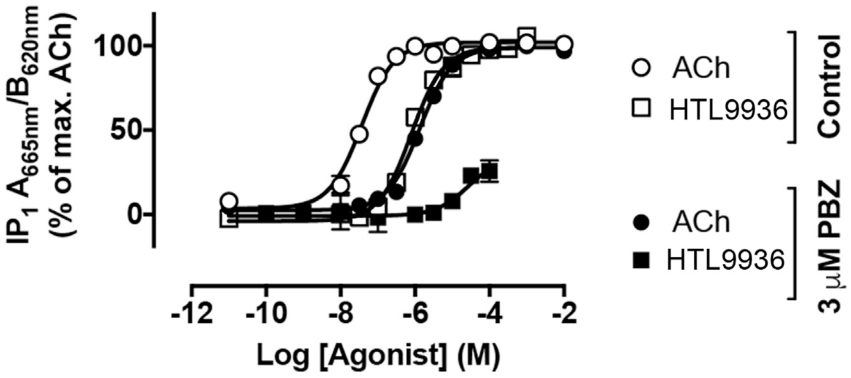

Figure S4. Partial agonist activity of HTL9936, related to Figure 4G

Inositol phosphate accumulation elicited by ACh or HTL9936 under control conditions or following pre-incubation (30 min) with 3 μM phenoxybenzamine to irreversibly reduce receptor expression in CHO Flp-In cells expressing the human M_1-receptor. Data are means ± S.E.M. of 3-4 independent experiments performed in duplicate.

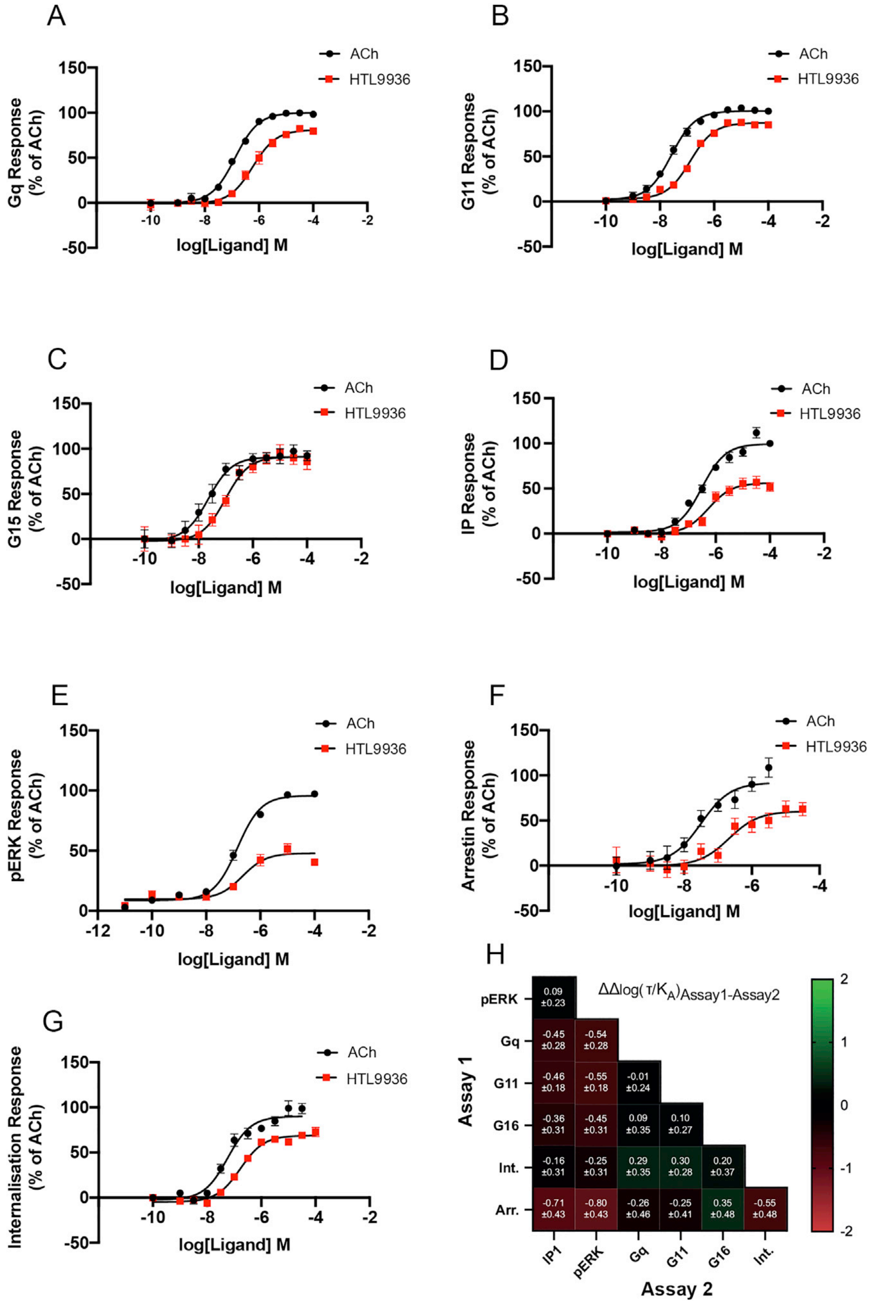

Figure S5. HTL9936 is an unbiased partial agonist of the M1-receptor, related to Figure 4G

A-G. Concentration responses are shown for ACh and HTL9936 using assays assessing a variety of M1-receptor signalling pathways and/or activation readouts. All data are shown as mean ± SEM and presented as percentages of the maximal ACh response from the same assay. A-C. Responses in BRET biosensor assays measuring activation of Gq, G11 or G15 G proteins. N=5, performed in triplicate. D. Accumulation of IP1 in response to 1 h treatment, N=5 in quadruplicate. E. Levels of phosphoERK induced in response to 5 min treatment. N=3 performed in duplicate. F. Recruitment of arrestin to the cell membrane as assessed using a bystander BRET assay, N=6 in quadruplicate. G. Internalisation of the M1-receptor, assessed through a bystander BRET assay measuring translocation of M1 receptors to early endosomes. N=4, in quadruplicate. H. Summary of ΔΔlog(τ/KA) calculated based on the data presented in A-G. All ΔΔlog(τ/KA) values are between 1 and -1, indicating that HTL9936 performed as a relatively unbiased agonist of the M1 receptor.

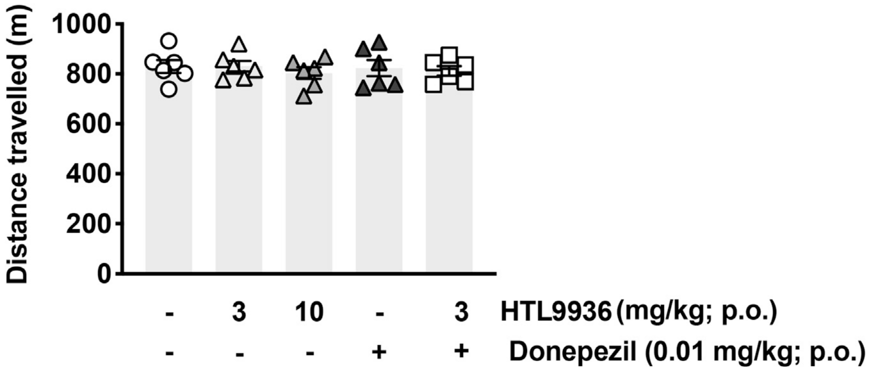

Figure S6. Effects of acute administration of HTL9936 and the cholinesterase inhibitor donepezil on open field exploratory behaviour, related to Figure 5B

Data are means ± S.E.M. of 6 rats and show distance travelled (cm) over a 5 minute period.

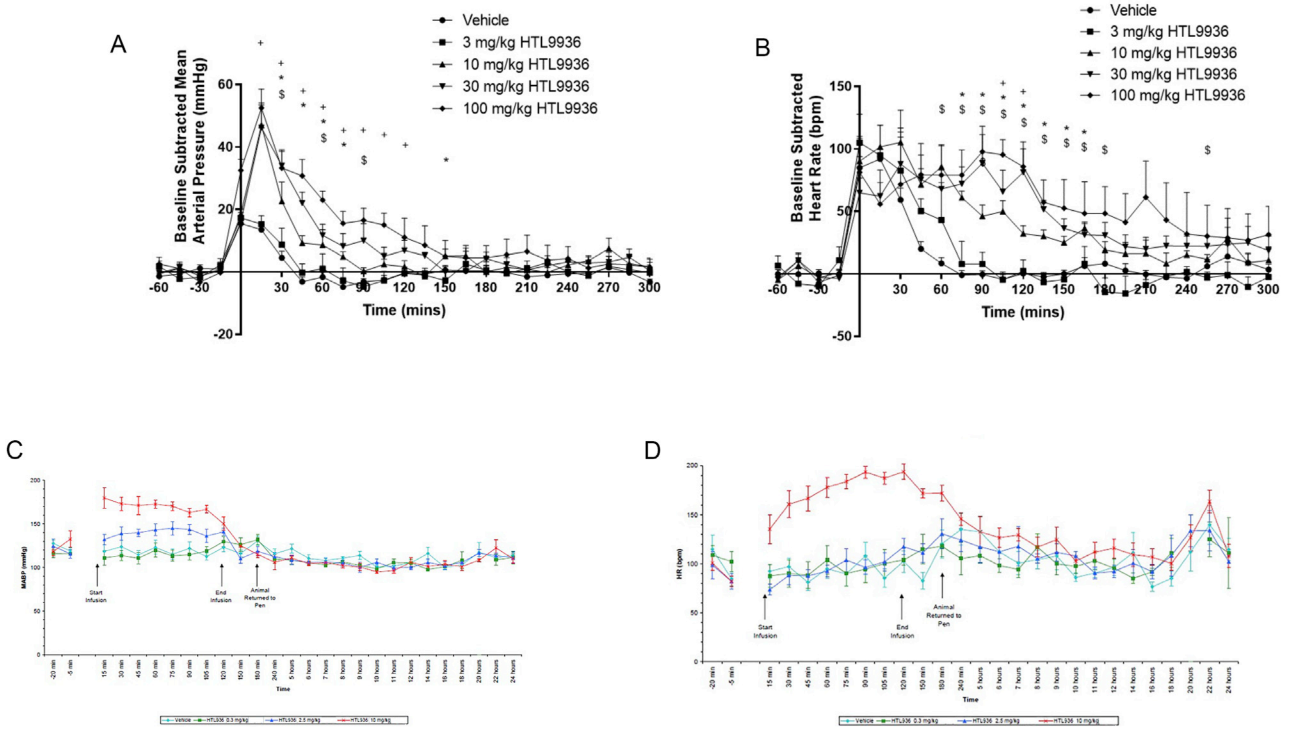

Figure S7. Effects on heart rate and blood pressure of intravenous administration of HT9936 in rats and dogs, related to STAR Methods

A. Averaged baseline subtracted mean arterial blood pressure effects in rats across over −60 to 300 minute time period. HTL9936 was administered at time = 0. Data are expressed as mean ± S.E.M. analysed by repeated measurement ANOVA and pairwise comparisons of each compound to vehicle by post-hoc Dunnett's test. Significant values are relative to the vehicle treatment only. Differences were considered to be significant by $P < 0.05$ for 10 mg/kg $^{\$}$, 30 mg/kg * and 100 mg/kg $^{+}$ respectively. **B.** Averaged baseline subtracted heart rate effects in rats across over −60 to 300 minute time period. HTL9936 was administered at time = 0. Data are expressed as mean ± S.E.M. analysed by repeated measurement ANOVA and pairwise comparisons of each compound to vehicle by post-hoc Dunnett's test. Significant values are relative to the vehicle treatment only. Differences were considered to be significant by $P < 0.05$ for 10 mg/kg $^{\$}$, 30 mg/kg * and 100 mg/kg $^{+}$ respectively. **C.** Group mean effects on mean atrial blood pressure in dogs. Data presented as mean ± S.E.M. (n=6 except ghoup 1 n=5 at 240 mins, group 2 n=0 at 20 hours, n=3 at 22hours and 24 hours, group 4 n=3 at 240 mins, n=4 at 5 hours and n=5 at 16 hours. **D.** Group mean effects on heart rate in dogs. Data presented as mean ± S.E.M. (n=6 except ghoup 1 n=5 at 240 mins, group 2 n=0 at 20 hours, n=3 at 22hours and 24 hours, group 4 n=3 at 240 mins, n=4 at 5 hours and n=5 at 16 hours.
